# Bismuth-Based Materials as Solar-Driven Photo(Electro)Catalysts for Environmental Remediation

**DOI:** 10.3390/ma19040728

**Published:** 2026-02-13

**Authors:** Muhammad Ashraf, Jiang Guo, Kai Yan, Jingdong Zhang

**Affiliations:** Key Laboratory of Material Chemistry for Energy Conversion and Storage (Ministry of Education), Hubei Key Laboratory of Bioinorganic Chemistry & Materia Medica, School of Chemistry and Chemical Engineering, Huazhong University of Science and Technology, Luoyu Road 1037, Wuhan 430074, Chinayank@hust.edu.cn (K.Y.)

**Keywords:** photoelectrocatalysts, photocatalysts, photoactive materials, bismuth-based semiconductors, environmental remediation

## Abstract

Bismuth-based semiconductors have emerged as a promising class of visible-light-responsive photo(electro)catalysts for environmental remediation owing to their tunable electronic structures, moderate band gaps, and relatively low toxicity. The stereochemically active Bi^3+^ 6s^2^ lone pair and strong Bi–O orbital hybridization tailor valence-band states, enabling enhanced utilization of the solar spectrum and favorable charge-carrier dynamics. In addition, layered, perovskite-like, and aurivillius-type crystal frameworks generate internal electric fields that are advantageous for photoelectrochemical (PEC) operation. This review critically examines advances from 2015 to 2025 in the design, synthesis, modification, and environmental applications of bismuth-based photo(electro)catalysts, with particular emphasis on PEC systems for pollutant degradation. Major material families, including bismuth oxides, oxyhalides, oxychalcogenides, chalcogenides, perovskite-like oxides, and complex metal oxides, are discussed in relation to their structure–property–performance relationships. Key synthesis strategies, such as solid-state, sol–gel, hydro/solvothermal, microwave-assisted, spray pyrolysis, and electrodeposition methods, are compared with respect to morphology control, defect chemistry, and electrode integration. Performance-enhancing approaches, including elemental doping, oxygen-vacancy engineering, and the rational design of type-II, p–n, Z-scheme, and S-scheme heterojunctions, are critically assessed. Practical considerations related to stability, scalability, and techno-economic constraints are highlighted. Finally, current challenges and future directions toward durable and application-ready bismuth-based PEC technologies are outlined.

## 1. Introduction

**Figure 1:**
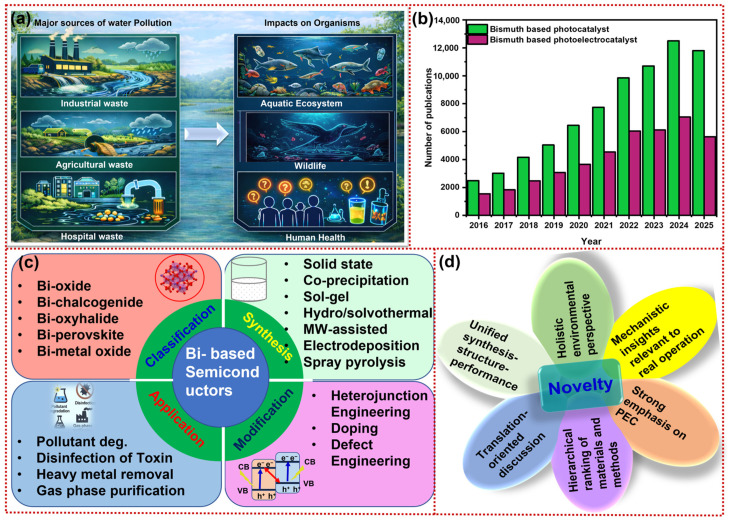
(**a**) Major sources of water pollution and pathways of contaminant release into aquatic environments, (**b**) publication trends for bismuth-based photocatalysts and photoelectrocatalysts from 2016 to 2025 (Google Scholar search, accessed 8 December 2025), (**c**) scope and structure of this review, (**d**) conceptual illustration highlighting the novelty and decision-oriented focus of the review.

Rapid industrialization, population expansion, and accelerating urbanization have intensified global pressure on water and energy resources. As a result, environmental quality and public health are increasingly threatened by the continuous discharge of complex pollutants into aquatic environments [1]. As illustrated in Figure 1a, emerging contaminants, including pharmaceuticals, pesticides, dyes, endocrine-disrupting compounds, and industrial chemicals, are increasingly being detected in natural water systems. These enter the environment via domestic discharges, industrial effluents, agricultural runoff, and atmospheric deposition, and their persistence and transformation in aquatic environments pose multifaceted ecological risks, ranging from microorganisms to higher trophic levels [2,3]. Despite the growing complexity of pollutant streams, global wastewater treatment capacity remains critically insufficient. Global assessments indicate that the domestic sector produces approximately 267.5 billion m^3^ of wastewater annually; of this volume, about 63% (168.8 billion m^3^ yr^−1^) is captured through sewer networks or septic systems, while only 54.7% (146.3 billion m^3^ yr^−1^) undergoes treatment before discharge. Alarmingly, the remaining 45.3% (121.2 billion m^3^ yr^−1^) is released directly into the environment without treatment, comprising both wastewater that is never collected (98.7 billion m^3^ yr^−1^) and wastewater that is collected but inadequately treated (22.5 billion m^3^ yr^−1^). These indicators underscore the pressing need for water purification technologies that effectively remove emerging pollutants [4].

Conventional water-treatment technologies, including coagulation, adsorption, membrane filtration, biological treatment, and electrochemical processes, are widely used but often exhibit limitations such as incomplete removal of persistent contaminants, high energy demand, secondary waste generation, membrane fouling, and high cost. In particular, they are sometimes ineffective in removing emerging contaminants, such as pharmaceuticals, antibiotics, and endocrine-disrupting compounds. Thus, the need for sustainable solar-driven technology, namely photocatalysis (PC), capable of degrading pollutants under mild conditions, is desired [5]. Furthermore, photoelectrochemical (PEC) systems that combine PC with electrochemistry can achieve superior charge separation by applying an external bias, directing photogenerated electrons and holes (h^+^) to spatially separated reaction sites. This bias-assisted charge management suppresses recombination losses, enhances reactive oxygen species (ROS) generation, and enables pollutant mineralization. Moreover, PEC processes typically consume less electrical energy than purely electrochemical treatments and avoid catalyst recovery issues associated with slurry PC, highlighting their practical potential for wastewater remediation [6,7]. Nevertheless, the efficiency of the PEC system critically depends on the properties of the photoactive semiconductor, including its light absorption, charge-transport behavior, stability, and band-edge alignment.

Early photo(electro)catalytic systems were primarily based on wide-bandgap semiconductors such as TiO_2_ and ZnO, which are activated by ultraviolet (UV) irradiation. However, UV light accounts for only a small fraction of the solar spectrum, resulting in low solar energy utilization [8]. In this context, visible-light-responsive semiconductors are particularly desirable, as they can harness the dominant portion of the solar spectrum while maintaining sufficient redox potential for pollutant degradation [9]. Among the various visible-light-responsive semiconductors explored for photo(electro)catalytic applications, bismuth-based materials have received particular attention due to their combination of optical tunability, favorable electronic structure, and intrinsic environmental stability [10]. These materials exhibit band gaps across the UV and visible range, such as 2.4 eV for monoclinic BiVO_4_, 1.6–1.9 eV for BiOI, and 3.1–3.5 eV for BiOCl, enabling efficient solar photon absorption. Visible-light absorption can be further enhanced by nanoscale architectures, such as Bi quantum dots, which promote efficient electron-hole (e-h) generation [10]. The optoelectronic behavior of bismuth-based semiconductors arises from the hybridization of Bi 6s and O 2p orbitals, which introduces additional electronic states near the V_B_ maximum. This interaction narrows the band gap and improves photoexcitation, light absorption, and carrier mobility. The stereochemically active Bi 6s lone pair induces structural distortions in Bi–O or Bi–X frameworks, creating internal dipole fields that promote directional charge separation, which is crucial for high PEC performance [11,12,13]. This trend aligns with the rapid growth in scientific publications on bismuth-based photo(electro)catalysts reported from 2016 to 2025 (Figure 1b). Together with their environmental stability and low toxicity (e.g., BiVO_4_, Bi_2_O_3_, and BiOX), these attributes position bismuth-based semiconductors as remarkable and environmental friendly materials for PEC water purification.

This review presents a mechanistically grounded synthesis–structure–performance framework for bismuth-based photo(electro)catalysts. It explicitly links fabrication routes to defect chemistry, crystal evolution, morphology, and band-structure modulation. These structure descriptors are then correlated with the photo(electro) catalysts’ degradation performance. In addition to surveying materials and strategies, it provides a deeper mechanistic discussion of oxygen-vacancy stability, halide leaching, interfacial electric fields, and S/Z-scheme charge-transfer behavior, with a focus on PEC-relevant factors such as photocurrent response, film microstructure, charge-transport interfaces, and device-integration challenges. Rather than a descriptive survey, this review serves as a decision-making framework and design roadmap. It enables the rational selection of Bi-based materials, synthesis routes, and modification strategies to meet targeted photo(electro)catalytic performance requirements. The review further broadens the scope across both aqueous and gas-phase remediation and incorporates translation-oriented considerations (scale-up, durability in real matrices, techno-economics, and life-cycle impacts). The main content and novelty of the review are shown in Figure 1c,d.

Previous reviews on bismuth-based photocatalysts have mainly focused on cataloguing material families and modification strategies, typically summarizing Bi-based oxides, oxyhalides, sulfides and vanadates in terms of crystal structure, band gaps, and generic photocatalytic activity for environmental remediation or solar energy conversion, with emphasis on dopants, heterojunction construction, and reaction pathways under unbiased photocatalytic conditions [14,15,16]. This review offers a decision-oriented application-driven perspective, with particular focus on photoelectrochemical (PEC) systems for environmental remediation. It integrates material classification, synthesis strategies, heterojunction engineering, and mechanistic insights with practical considerations, including operating conditions, stability, real water matrices, and scalability. By critically linking structure–property–performance relationships under realistic PEC conditions, this review aims to provide clearer guidance for material design and system development, addressing current research trends and practical implementation challenges.

More recent reviews (2023–2025) have narrowed their scope toward specific sub-classes or applications, such as BiVO_4_ photoanodes for PEC water splitting [17], BiOX-based photocatalysts [18], for water purification, or defect/heterojunction engineering to enhance charge separation [19], but these studies largely remain material-centric, with limited cross-comparison among different Bi families, insufficient attention to electrode fabrication, bias-induced stability, and durability under PEC operation, and only a brief discussion of real-matrix or scale-up constraints. In contrast, this review is novel in that it adopts a PC as well as PEC-centered, application-oriented framework across multiple bismuth material families, explicitly linking synthesis routes to defect chemistry, band modulation, film microstructure, and interfacial charge transport, while critically evaluating stability, degradation mechanisms, and scalability under realistic PEC conditions, thereby moving beyond descriptive surveys to provide a design-oriented roadmap for translating Bi-based photo(electro)catalysts from laboratory studies to practical environmental remediation systems.

## 2. Classification of Bismuth-Based Materials

### 2.1. Bismuth Oxide

**Figure 2:**
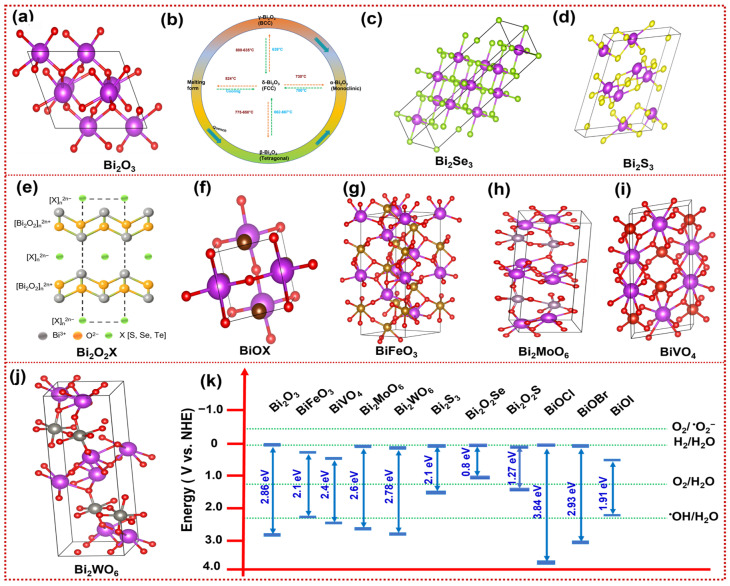
Representative crystal structures of bismuth-based semiconductors: (**a**) Bi_2_O_3_, (**b**) phase transformation relationships among Bi_2_O_3_ polymorphs, (**c**) Bi_2_Se_3_, (**d**) Bi_2_S_3_, (**e**) Bi_2_O_2_X (X = S, Se, Te), (**f**) BiOX (X = Cl, Br, I), (**g**) BiFeO_3_, (**h**) Bi_2_MoO_6_, (**i**) BiVO_4_, (**j**) Bi_2_WO_6_ and (**k**) schematic band-structure alignment of typical Bi-based semiconductors.

Bismuth oxides (Bi_2_O_3_), due to their crystal structure (Figure 2a) and better photocatalytic properties, are ideal candidates for various environmental and energy-related applications. Bi_2_O_3_, a p-type semiconductor, has a suitable band gap of 2–3.9 eV. The photocatalytic activity of Bi_2_O_3_ is enhanced by natural polarization and hybridization, thereby improving the separation of photogenerated charge carriers [11,12]. Bi_2_O_3_ nanoparticles generate high levels of ROS upon exposure to visible light. These ROS enable the oxidative degradation of organic pollutants upon adsorption onto the nanoparticle surface. However, Bi_2_O_3_ is hindered by e-h recombination and unstable crystal phases, which limit its long-term photocatalytic performance. As Bi_2_O_3_ exists in four polymorphic forms (Figure 2b), among these, α-Bi_2_O_3_ with a monoclinic phase is more stable at ambient conditions, and δ-Bi_2_O_3_ with a face-centered cubic structure is typically stabilized at an elevated temperature or via doping/defect stabilization. β-Bi_2_O_3_ and γ-Bi_2_O_3_ are metastable and tend to convert into α-Bi_2_O_3_ under higher temperatures [20,21]. Despite this limitation, its relatively low toxicity and good photocatalytic performance make it an attractive material for pollutant degradation.

### 2.2. Bismuth Chalcogenides

From a materials-design perspective, beyond oxide-based systems, bismuth chalcogenides, particularly Bi_2_Se_3_ [22] and Bi_2_S_3_ [23], have emerged as an essential class of narrow-bandgap semiconductors for visible-light PEC. Both Bi_2_Se_3_ and Bi_2_S_3_ are n-type semiconductors with unique crystal structures, as illustrated in Figure 2c,d, and narrow band gaps, making them promising candidates for environmental applications [24,25].

Bi_2_Se_3_ exists in two distinct crystal phases: the thermodynamically stable rhombohedral phase, with a narrow band gap of approximately 0.3 eV, is particularly advantageous for thermoelectric applications [26]. The metastable orthorhombic phase, with an approximately 1.2 eV band gap, exhibits electronic properties advantageous for PEC applications. This phase transition results in significant differences in electrical conductivity, with the rhombohedral phase exhibiting higher conductivity. The rhombohedral phase of Bi_2_Se_3_ has a quintuple-layer structure, while the orthorhombic phase contains distorted [BiSe_6_] octahedra. These structural features give rise to its unique electronic and optical properties [26]. Bi_2_S_3_ has a narrow band gap, making it suitable for photo(electro)catalytic applications; its structure offers a high surface area, abundant active sites, and improved interfacial kinetics, thereby enhancing performance [27]. Surface modifications, doping, and heterostructure formation have been explored to optimize charge-carrier dynamics and pollutant-degradation activity.

### 2.3. Bismuth Oxychalcogenides (Bi_2_O_2_X)

In addition to chalcogenide systems, bismuth oxychalcogenides (B_2_O_2_X) (n-type semiconductors) such as Bi_2_O_2_S, Bi_2_O_2_Se, and Bi_2_O_2_Te constitute a rapidly growing family of layered semiconductors with promising properties for visible-light absorption and PEC applications. B_2_O_2_X materials possess a distinctive quasi-2D layered structure (Figure 2e) in which [Bi_2_O_2_]^2+^ layers alternate with chalcogen X layers (X = S, Se, Te), imparting moderate band gaps, high electron mobility, and excellent environmental stability. First-principles and experimental studies report that these materials generally exhibit size-tunable band gaps in the range of ~0.8–1.27 eV, which is favorable for visible-light harvesting, and high carrier mobility due to their non-van der Waals bonding and low effective mass [28].

Bi_2_O_2_Se, in particular, has attracted significant research attention as a novel quasi-2D semiconductor, exhibiting high ambient stability, a tunable band structure, and strong charge-transport behavior [29]. Although much of the work on Bi_2_O_2_X materials has focused on electronics and photodetection, recent research has begun exploring their photocatalytic responses. For example, high-quality epitaxial Bi_2_O_2_S films have demonstrated a strong visible-light photoresponse, with photoresponsivity of ~60 mA W^−1^ and on/off ratio of ~10^4^ under red-light illumination, indicating efficient absorption and carrier separation in the visible regime [30]. Moreover, alloying strategies in quasi-2D Bi_2_O_2_(S_X_Se_1−X_) epitaxial films have shown that field-effect mobility and electronic properties can be systematically tuned by composition, with measured electron mobilities exceeding ~215 cm^2^ V^−1^ s^−1^ for a Bi_2_O_2_(S_0.4_Se_0.6_), which highlights the strong charge migration performance achievable through compositional alloying in epitaxial heterostructures [31].

These properties make bismuth oxychalcogenides a promising complement to Bi-oxides and Bi-chalcogenides, with heterojunction and defect engineering offering clear pathways to improved visible-light PEC performance for water treatment.

### 2.4. Bismuth Oxyhalides (BiOX)

From a crystal-structure standpoint, bismuth oxyhalides, including BiOCl [32], BiOBr [33], and BiOI [34], differ fundamentally from oxides and chalcogenides due to their [Bi_2_O_2_]^2+^ slabs (Figure 2f), with a strong influence on charge transfer and surface reactivity. BiOX often behaves as an n-type (or sometimes p-type) semiconductor, depending on defect chemistry and synthesis conditions, and on band gaps, which are crucial for its application under visible-light irradiation [35]. BiOCl, BiOBr, and BiOI possess band gaps of approximately 3.9–3.5 eV, 2.92 eV, and 1.90 eV, respectively, thereby enabling effective photocatalytic degradation of organic pollutants [36]. The layered structure not only imparts structural flexibility but also prolongs carrier lifetime upon light absorption [37]. The conduction band (C_B_) is primarily composed of Bi 6p orbitals, whereas the VB is dominated by halogen np and oxygen 2p orbitals, resulting in a favourable band structure for photocatalytic applications [38]. Among BiOX materials, BiOI exhibits favorable PEC performance due to its narrow band gap, which facilitates visible-light absorption. However, these materials are prone to charge recombination, which hinders their photocatalytic activity [39]. To mitigate this, strategies such as doping, surface modification, and heterojunction formation are employed to enhance charge transfer, broaden the absorption spectrum into the visible range, and enhance overall photocatalytic performance.

### 2.5. Bismuth-Based Perovskite-like Oxides

From a structural engineering perspective, perovskite-like bismuth oxides offer additional flexibility through lattice distortion, ferroelectricity, and internal polarization. BiFeO_3_ belongs to the perovskite family (intrinsically p-type, but defect engineering can turn it n-type); its perovskite crystal structure (Figure 2g) provides both structural stability and reduction in charge recombination [40,41,42]. The electronic structure of BiFeO_3_ contributes to its effective PEC performance, with a relatively narrow band gap (2.0–2.2 eV), thereby enabling activation under visible light [40]. The V_B_ consists of oxygen 2p and iron 3d orbitals, while the C_B_ is primarily dominated by iron 3d states, hybridized with bismuth 5p orbitals. This hybridization improves electron transfer, which is crucial for high photocatalytic activity and charge mobility [40,43]. Strategies such as doping with lanthanum (La) or manganese (Mn) and the formation of heterojunctions have been employed to minimize recombination and enhance performance [44,45]. Additionally, morphologies such as nanoparticles, nanofibers, and thin films have been used to increase surface area and improve PEC activity.

### 2.6. Complex Bismuth-Based Metal Oxides

Bismuth-based metal oxides are n-type semiconductors and constitute another critical class of photo(electro)catalysts, characterized by well-defined crystal structures, tunable band gaps, and good chemical stability for environmental remediation applications. Bismuth Molybdate (Bi_2_MoO_6_), a member of the aurivillius oxide family with a band gap around 2.6 eV, exhibits a unique structure, as shown in Figure 2h, where [Bi_2_O_2_]^2+^ layers alternate with MoO_6_ perovskite layers. This arrangement enables excellent visible-light absorption and makes Bi_2_MoO_6_ an effective candidate for the degradation of organic pollutants, water splitting, and CO_2_ reduction [21,46]. Bismuth Vanadate (BiVO_4_), in particular, monoclinic scheelite BiVO_4_ (m-BiVO_4_), is one of the most widely studied bismuth-based semiconductors [47], as shown in Figure 2i. It exhibits favorable light absorption capability, favorable band-edge positions, and high electrochemical stability. With a band gap of approximately 2.4 eV, BiVO_4_ can absorb visible light effectively, and its C_B_ is composed of V 3d orbitals. In contrast, the V_B_ arises from the hybridization of Bi 6s and O 2p orbitals [47,48]. Bismuth Tungstate (Bi_2_WO_6_) exhibits a wider band gap (~2.8 eV) as compared to that of BiVO_4_ and Bi_2_MoO_6_. The crystal structure of Bi_2_WO_6_, as depicted in Figure 2j, consists of alternating [Bi_2_O_2_]^2+^ and [WO_4_]^2−^ layers, generating numerous adsorption sites and producing a built-in electric field that aids in the separation of photogenerated e-h pairs [49,50]. However, like Bi_2_MoO_6_ and BiVO_4,_ Bi_2_WO_6_ suffers from rapid e-h recombination. Strategies such as morphology control, metal doping, and heterojunction formation have been employed to improve their photocatalytic performance. The band gap structures of bismuth-based semiconductors are shown in Figure 2k. A summary of bismuth-based materials is provided in Table 1.

Having outlined the prominent Bi-based material families and their structure–property features, the following section compares synthesis routes. These routes control morphology, defect density, and interfacial architecture, which ultimately govern PEC performance.

## 3. Synthesis Strategies of Bi-Based Materials

### 3.1. Solid-State Method

The solid-state method is among the earliest and most widely used routes for synthesizing bismuth-based oxides and mixed compounds. In this technique, precursor powders such as Bi_2_O_3_ and V_2_O_5_ are intimately ground and calcined at 500–800 °C to obtain the desired crystalline phases. This approach is solvent-free, cost-effective, and highly scalable, making it attractive for bulk synthesis [49,51].

**Figure 3:**
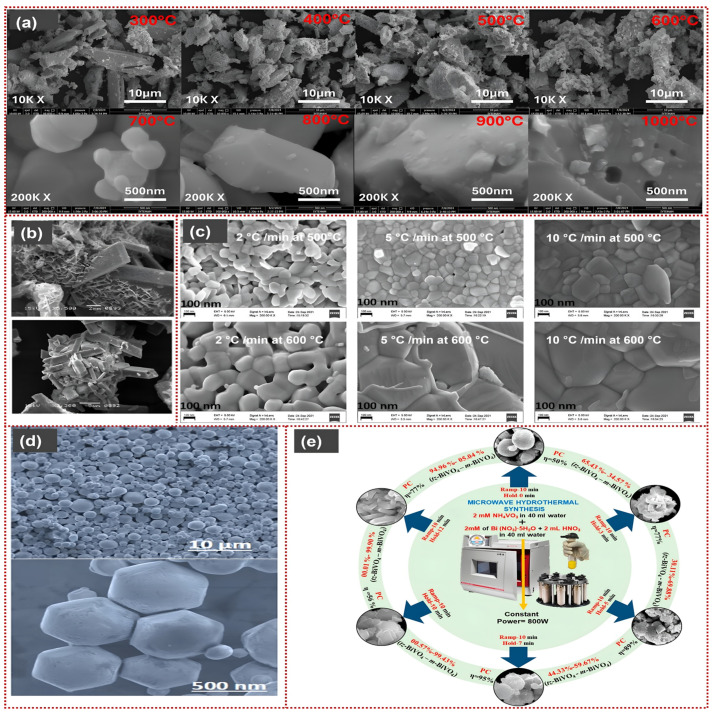
Morphological and structural evolution of Bi-based materials synthesized via different routes: (**a**) SEM images of Bi_2_WO_6_ prepared at different calcination temperatures. Reproduced with permission from [52], *International Journal of Applied Ceramic Technology*, © 2025 the Author(s), published by Wiley Periodicals LLC on behalf of the American Ceramic Society, licensed under CC BY-NC-ND. (**b**) SEM images of Bi_2_O_3_/MgO/GO synthesized by the co-precipitation method. Reproduced from [54], *RSC Advances*, 2022, © the Author(s), licensed under CC BY-NC 3.0 (**c**) SEM images of sol–gel-derived BiFeO_3_ nanoparticles annealed at different temperatures and heating rates. Reproduced from [58], IOP *Conference Series: Materials Science and Engineering*, 2022, © the Author(s), licensed under CC BY 3.0 (**d**) SEM micrographs of m-BiVO_4_ prepared by the hydrothermal method. Reproduced from [62], *Scientific Reports*, 2020, © the Author(s), licensed under CC BY. (**e**) Schematic illustration of the MW-HT synthesis of BiVO_4_ catalysts with different crystalline phases and morphologies. Reprinted with permission from [79]. Copyright © 2019 American Chemical Society.

Kayhan et al. reported that heating Bi_2_O_3_ and WO_3_ below 300 °C does not induce Bi_2_WO_6_ formation; X-ray Diffraction (XRD) showed mainly unreacted oxide phases [52]. At 400–500 °C, a phase transition to oxygen-deficient Bi14W2O27 occurs, accompanied by the lowest band gap (2.63 eV). This narrowing band gap is consistent with increased oxygen-vacancy content and electronic interactions at moderate temperatures. The scanning electron microscope (SEM) shows nanoscale facets and partially ripened cubic platelets in the 400–500 °C samples. These nanoscale features increase active surface exposure and improve light harvesting. At elevated temperatures of 600–700 °C, the phase progressively converts to orthorhombic Bi_2_WO_6_, accompanied by grain growth and agglomeration into micron-sized crystals. Above 800 °C, only highly crystalline Bi_2_WO_6_ remains, with the loss of nanoscale features critical for optical activity (Figure 3a). The elimination of oxygen-deficient phases at high temperatures widens the band gap (~2.82 eV), reducing visible-light response. Samples treated at 400–500 °C exhibit the strongest photoactivity; this temperature range balances defect density, band-gap narrowing, and morphology. In contrast, excessive heating removes oxygen vacancies (O_v_) and induces dense grain growth, which diminishes activity [52].

Vu et al. reported the synthesis of BiVO_4_ via a solid-state reaction between BiOI and NH_4_VO_3_ at 400 °C, revealing that both calcination time and precursor ratio critically govern performance. Extended calcination (10–12 h) enhances crystallinity but also increases particle size, which can limit activity. An equimolar BiOI/NH_4_VO_3_ ratio yields phase-pure BiVO_4_ with optimal stability, whereas excess BiOI or NH_4_VO_3_ introduces Bi_2_O_3_ or V_2_O_5_ impurities, respectively. The sample calcined for 10 h exhibited the highest photocurrent density (0.25 mA cm^−2^), highlighting the need for precise control of solid-state synthesis parameters [53]. Overall, while solid-state reaction (SSR) is scalable and straightforward, it is generally unsuitable for producing nanosized Bi-based materials due to the high temperatures required (≈400–700 °C), which promote sintering, lead to large grain size, reduce surface area, and make purity and morphology difficult to control [16].

### 3.2. Co-Precipitation

The inherent drawbacks of solid-state synthesis, including large particle sizes, poor dispersion, and limited control over morphology, have driven interest in solution-based routes, such as co-precipitation. Chemical precipitation from homogeneous aqueous media offers a scalable and economical strategy for preparing Bi-based semiconductors. Compared with high-temperature solid-state methods, co-precipitation operates under milder conditions, reducing energy consumption and costs, and enabling precise control over solution chemistry, typically producing finer particles with higher specific surface areas [16]. Co-precipitation remains a versatile route for synthesizing bismuth oxyhalides (BiOX), in which solution acidity and halide coordination govern crystal growth and facet exposure. Urooj et al. prepared a Bi_2_O_3_/MgO/GO ternary nanocomposite via a co-precipitation method followed by low-temperature annealing. SEM images (Figure 3b) showed uniform dispersion of MgO nanoparticles on GO sheets, while Bi_2_O_3_ forms larger plate-like crystallites. The intimate interfacial contact among the components confirms the formation of a well-integrated heterostructure, which is favorable for efficient carrier transport in PC and PEC applications [54]. Similarly, Wu et al. reported that CTAB-assisted precipitation under acidic conditions produces plate-like BiOBr. At the same time, precise control of the final acidity enables selective exposure of either the (001) or the (010) facets. BiOBr-(010) showed higher photo-oxidative activity than BiOBr-(001). This is reflected in higher oxygen-evolution rates and faster formic-acid degradation rates [55].

Compared with solid-state synthesis, co-precipitation enables lower-temperature processing and partial control of morphology; however, particle agglomeration and limited tunability of defects still constrain its photo(electro)catalytic performance [16].

### 3.3. Sol–Gel Method

Sol–gel is simple, inexpensive and can offer better compositional homogeneity and morphology control than co-precipitation [56,57]. Eledath et al. reported the synthesis of BiFeO_3_ nanostructures via a sol–gel route in an ethylene glycol/acetic acid medium, followed by gelation at 100 °C. Annealing at 500–600 °C yields 100–500 nm particles; the resulting band gaps fall around 2.18–2.26 eV. Higher annealing temperatures enhance BiFeO_3_ crystallinity. Careful control of pH, calcination time and temperature yields homogeneous rhombohedral BiFeO_3_ powders with tunable grain size, as shown in Figure 3c, and improved photocatalytic performance [58]. Santiago et al. reported Bi_2_MoO_6_ synthesis using Bi(NO_3_)_3_·5H_2_O, ammonium molybdate, citric acid, and PEG-200 to form a polymeric blue gel, which was calcined at 450–500 °C to obtain Bi_2_MoO_6_/Bi_6_Mo_3_O_15_ composites. These samples exhibited a band gap of 2.97 eV (reduced to 2.9 eV after Au decoration), achieving sound methylene blue degradation and CO_2_ photoreduction [59]. Akşit et al. reported that sol-gel synthesis enables effective control of the morphology of Bi_2_O_3_ xerogels. Calcination of bismuth acetate–oxalic acid ethanol sols at 400 °C produces flower-like β-Bi_2_O_3_ nanoparticles. These materials exhibit small crystallite sizes (~31 nm), high surface areas (~23 m^2^g^−1^), and rapid sunlight-driven photoactivity [60].

Compared with co-precipitation, sol–gel routes provide higher compositional, finer particle dispersion, and improved control over band structure and defect chemistry, resulting in significant visible-light photocatalytic activity.

### 3.4. Hydro/Solvothermal Method

Although sol–gel methods are effective for nanoparticle synthesis, their practical scalability is limited by slow processing, particularly during gelation and drying. In addition, gel shrinkage and cracking during drying can compromise particle uniformity and quality, posing challenges for large-scale production [61]. Hydro/solvothermal methods often provide stronger morphological control and yield a more uniform particle-size distribution, as shown in Figure 3d [62]. They also deliver higher crystallinity under relatively mild conditions [63,64]. Both morphology and crystallinity strongly influence pollutant removal by PC and PEC. Reaction parameters such as pH, temperature, solvent type, and reaction time are critical for tuning the physicochemical characteristics of the resulting materials [65,66,67]. For instance, Bi_2_MoO_6_ synthesized at low pH (2–4) tends to form nanoplates or nanosheets, while higher pH (6–10) favors spherical nanoparticles. The nanosheet morphology obtained at lower pH values exhibit a higher surface area and more exposed active facets, leading to increased degradation of RhB and phenol [68,69,70].

Temperature also plays a critical role during hydrothermal synthesis. For bismuth oxide formate (BiOCOOH), heating between 100 and 160 °C yields well-ordered, flower-like structures with optimal photocatalytic performance. Higher temperatures damage the crystal structure, narrow the band gap to ~3.40 eV, and reduce performance. In NaBiO_3_ systems, higher synthesis temperatures improve crystallinity and increase particle size. A phase transition from NaBiO_3_·nH_2_O to ilmenite-type NaBiO_3_ occurs at around 232 °C. Elevated temperatures also promote O_V_ formation, thereby enhancing photocatalytic activity [66,71]. Liu et al. reported that temperature strongly affects the formation of the BiVO_4_ phase during hydrothermal synthesis. At 120 °C for 1.5h, a single-phase P-BiVO_4_ was obtained. Upon heating to 200 °C for 1.5h, rod-like R-BiVO_4_ forms with a distinct two-phase structure. The two-phase material exhibits higher PEC degradation performance than its single-phase counterpart [72]. Zai et al. reported I-doped Bi_2_O_2_CO_3_ synthesis by the hydrothermal method from sodium citrate (Na_3_Cit), bismuth nitrate pentahydrate (Bi(NO_3_)_3_·5H_2_O), and sodium iodide as an iodine source at 180 °C for 24 h. The pH of the solution was adjusted by utilizing ammonium hydroxide. The material exhibited good photocatalytic activity. SEM analysis showed the rose-like morphology with a higher number of active sites [73].

Among wet-chemical methods, hydro/solvothermal synthesis offers the best control over crystal phase, exposed facets, and nanostructure morphology, which is particularly beneficial for prolonged charge lifetime and pollutant degradation. However, the long reaction time is the major limitation of this technique [74].

### 3.5. Microwave-Assisted Method

Microwave-assisted synthesis, including microwave hydrothermal (MW-HT) and solvothermal approaches, has emerged as a rapid and energy-efficient alternative. Microwave irradiation induces volumetric heating, accelerates nucleation and crystal growth, and shortens reaction times [75], often improving crystallinity and morphology. Several bismuth-based materials, including Bi_2_CrO_6_, Bi_2_S_3_, and Bi_2_Mo_3_O_12_, have been successfully synthesized via microwave-assisted methods and exhibit enhanced crystallinity. Previous studies have shown that the synthesis of Bi_2_CrO_6_ was carried out by the conventional method and the MW-HT method. Crystals produced by the microwave-assisted method exhibited higher crystallinity and a more uniform morphology than those produced by the conventional method [76,77,78]. Dabodiya et al. schematically illustrated the MW-HT synthesis strategy used to control the crystalline phase and morphology of BiVO_4_ by varying the microwave holding time at a constant power of 800 W. Figure 3e shows that short irradiation times favor the formation of tetragonal zircon-type BiVO_4_ microspheres. At the same time, prolonged holding induces dissolution-recrystallization and phase transformation into monoclinic scheelite BiVO_4_ with decahedral morphology. Intermediate holding times yield mixed monoclinic–tetragonal BiVO_4_ heterophases, in which the coexistence of both phases forms internal heterojunctions that significantly enhance photocatalytic efficiency [79]. Pattnaik et al. reported microwave-assisted synthesis of BiFeO_3_ nanoparticles that show high activity in PEC applications, achieving a 93.5% reduction in total organic carbon (TOC) from greywater within 180 min under moderate 50 W illumination [80]. Rodriguez-Giron et al. reported that α-Bi_2_Mo_3_O_12_ was successfully synthesized by MW-HT methods and used for the photocatalytic degradation of tetracycline [78].

These results illustrate how microwave-assisted synthesis can promote rapid crystallization, favourable defect structures, and improved light absorption, all of which enhance photocatalytic activity. Among the synthesis strategies discussed, microwave-assisted methods are particularly notable. These enable ultrafast crystallization, affordability, and the formation of defect-rich Bi-based nanostructures within minutes. These advantages make them highly attractive for the fabrication of next-generation photo(electro)catalysts.

### 3.6. Thin Film Formation Strategies

#### 3.6.1. Spray Pyrolysis

Spray pyrolysis is a versatile and industrially relevant method for depositing bismuth-based semiconductors. It offers precise control over aerosol atomization, film stoichiometry, and microstructure. During deposition, micrometer-scale droplets rapidly evaporate and decompose on heated substrates. This process yields uniform oxide films with tunable crystallinity and morphology, which are critical for PEC performance. Although challenges remain, such as low yield and temperature optimization, the method is suitable for scalable fabrication. Hernandez Simon et al. employed ultrasonic spray pyrolysis to prepare BiFeO_3_ hollow microspheres and BiFeO_3_–Bi_2_O_3_ flower-like structures. These architectures increased the short-circuit current density to 3.5 mA cm^−2^ and delivered an external quantum efficiency (EQE) of 10.7% at 431 nm [81]. Somdee et al. reported CuBi_2_O_4_ photocathodes, synthesized by water-based spray deposition followed by air annealing. This approach produced plate-like Kusachiite CuBi_2_O_4_ films with a band gap of 1.86 eV. The films delivered photocurrents of 0.2 mA cm^−2^ in Na_2_SO_4_ and 0.5 µA cm^−2^ in NaHCO_3_. Although the PEC response was modest, the method highlights simplicity and scalability [82].

Wakishima et al. developed a greener, carbon-free aqueous nitrate route for the synthesis of CuBi_2_O_4_ (CBO). The XRD results confirmed that the as-deposited film was largely amorphous, transforming into phase-pure tetragonal CBO only after post-annealing at ≥500 °C. Higher annealing temperatures (500–700 °C) progressively sharpened the characteristic CBO peaks, supporting good crystallinity and the elimination of residual Cu/Bi precursor phases. SEM revealed that the as-deposited film exhibited a porous, nonuniform morphology with ~500 nm protrusions and ~200 nm pores, whereas post-annealing yielded dense, pore-free surfaces with grain sizes increasing from ~200 to ~500 nm, consistent with the crystallite growth observed in XRD. Overall, annealing converts the initial porous deposit into a dense, well-crystallized CBO film with improved structural integrity [83]. Beyond thin films, flame spray pyrolysis (FSP) enables ultrafast droplet-to-particle conversion at higher flame temperatures. As reported by Li et al. Bi_4_Ti_3_O_12_/TiO_2_ heterostructures were synthesized in a single FSP step, showing higher photocatalytic activity under simulated sunlight due to effective Z-scheme charge separation [84].

**Figure 4:**
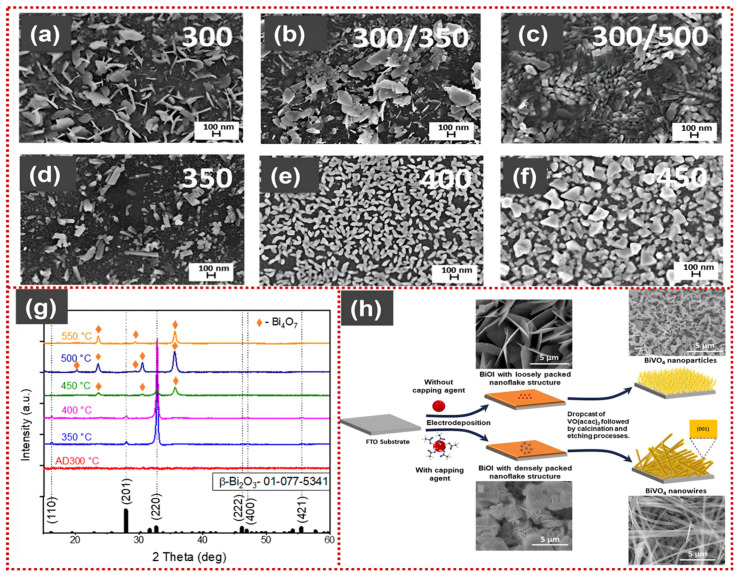
SEM images of Bi_2_O_3_ films at various conditions (**a**) grown at 300 °C without annealing, (**b**) grown at 300 °C and annealed at 350 °C, (**c**) grown at 300 °C and annealed at 500 °C, (**d**) grown at 350 °C without annealing, (**e**) grown at 400 °C without annealing, and (**f**) grown at 500 °C without annealing. Reproduced with permission from [85], *RSC Advances*, 2024, © the Author(s), licensed under CC BY-NC 3.0. (**g**) Corresponding XRD patterns of Bi_2_O_3_ films prepared by spray pyrolysis at different calcination temperatures. Reproduced with permission from [85], *RSC Advances*, 2024, © the Author(s), licensed under CC BY-NC 3.0. (**h**) Schematic of the electrodeposition-assisted route for morphology-controlled BiVO_4_ nanostructures derived from BiOI templates. Reproduced from [92], *Journal of Alloys and Compounds*, 2025, © the Author(s), licensed under CC BY.

Sydorenko et al. systematically investigated the effect of deposition and annealing temperature on the structure and morphology of spray-pyrolysed Bi_2_O_3_ thin films. SEM images (Figure 4a–f) show that films deposited at 300 °C exhibit irregular flake-like morphologies, while annealing at 350 °C results in a more compact flake-like structure. Higher annealing temperatures (≥500 °C) induce pronounced particle agglomeration, whereas increasing the deposition temperature from 350 to 450 °C results in a gradual transition to a granular morphology with enlarged grains [85]. XRD analysis (Figure 4g) reveals that the film deposited at 300 °C and annealed at 350 °C crystallizes into a pure β-Bi_2_O_3_ phase with a dominant (220) preferential orientation. In contrast, annealing at ≥450 °C leads to the formation of Bi_4_O_7_ secondary phases. The 300/350 °C condition provides an optimal balance among phase purity, crystallinity, and surface morphology, corresponding to the highest photocatalytic activity for methyl orange (MO) degradation reported in the study [85].

Collectively, these studies demonstrate that ultrasonic, pneumatic, and flame-assisted spray pyrolysis provide a tunable platform for constructing Bi-based PEC materials with controlled defect chemistry, scalable processing, and compatibility with multicomponent heterostructures. Despite the advantages of spray pyrolysis, its low yield impedes scale-up. Additionally, difficulties in determining the growth temperature limit its application [86].

#### 3.6.2. Electrodeposition Method

Electrodeposition offers precise control over film composition. It is low-cost, compatible with flexible substrates, and readily scalable. These advantages make it well-suited to thin-film fabrication [87], particularly for PEC applications. In this method, metal ions in solution are reduced and deposited onto conductive substrates under an applied potential, allowing precise control over film thickness, composition, and morphology. Recent studies have highlighted the potential of electrodeposited BiVO_4_ [88,89], BiOX, BiFeO_3_ [41], and Bi_2_Te_3_ [90] films for the PEC degradation of dyes, pesticides, and pharmaceuticals under visible light. These films, typically grown on fluorine-doped tin oxide (FTO) substrates, can adopt flower-like or nanowire architectures, thereby increasing the active surface area for pollutant adsorption [41,89]. Deposition time and voltage are critical parameters: short deposition durations (1–5 min) generally yield thin, smooth films with high charge-transport and improved PEC degradation rates [41,90], whereas longer times produce thicker films prone to bulk recombination and lower activity. Mohamed et al. reported BiVO_4_ nanostructured films via electrodeposition with controlled deposition times to tune the feature size. Short deposition times yielded smaller diameters (148.2–175.2 nm), whereas long deposition times resulted in pronounced growth with diameters of 332.3–449.7 nm. The finer nanostructures obtained at shorter deposition times are expected to offer a higher surface area, thereby promoting higher photocurrent density in PEC systems [91].

Kaur et al. developed a controlled electrodeposition–conversion strategy to fabricate BiVO_4_ nanostructures with tunable morphology and crystal facets for PEC water oxidation. As illustrated in Figure 4h, BiOI precursor films were first electrodeposited on FTO substrates, where the presence or absence of acetic acid as a capping agent dictated the packing density of BiOI nanoflakes. Loosely packed BiOI nanoflakes (without a capping agent) were converted into BiVO_4_ nanoparticles after drop-casting VO(acac)_2,_ followed by calcination and etching. In contrast, densely packed nanoflakes (with optimal acetic acid concentration) transformed into one-dimensional BiVO_4_ nanowires with preferentially exposed (001) facets. This morphology and facet control enabled the formation of a self-assembled BiVO_4_ nanoarchitecture, which significantly improved charge transport and photocurrent generation, highlighting the critical role of precursor packing and facet engineering in optimizing BiVO_4_ photoanodes [92].

Zheng et al. reported that electrodeposition parameters, including applied potential, deposition time, electrolyte pH, and precursor composition, critically govern the nucleation and growth behavior of BiVO_4_ films. By systematically varying the electrolyte pH, precursor sources, and deposition configuration, pristine BiVO_4_ (P-BiVO_4_), single-layer BiVO_4_ (SL-BiVO_4_), and bilayer BiVO_4_ (BL-BiVO_4_) photoelectrodes were successfully fabricated. SEM analysis reveals that P-BiVO_4_ exhibits a loosely packed nano-porous morphology with large interparticle voids, which hampers electron transport toward the Indium Tin Oxide (ITO) substrate. In contrast, SL-BiVO_4_ exhibits a more compact intimate packing structure, thereby favoring interfacial charge transfer. Notably, BL-BiVO_4_ consists of densely packed particle units with the highest structural compactness among the three configurations. This architecture markedly shortens the diffusion length of photogenerated charge carriers, thereby reducing bulk recombination [93]. Moderate deposition potentials (−0.1 to −0.6 V) typically yield smooth, compact films with directional carrier transport. In contrast, high overpotentials may generate rough surfaces and structural defects, which can increase photocurrents but also introduce recombination sites. BiVO_4_ films electrodeposited under optimized conditions exhibit higher photocurrent densities and higher pollutant degradation results [5,41,88,89].

Electrodeposition is a low-temperature, scalable, and cost-effective method for Bi-based films. It allows direct growth on conductive substrates without post-sintering. The films show good crystallinity, strong adhesion, and better carrier separation. These features are essential for stable PEC performance. Compared with powder routes, thin-film methods are more suitable for PEC applications. These enable direct integration with controlled thickness and charge-transport pathways. A summary of synthesis methods is provided in Table 2.

## 4. Photocatalytic Activities of Bismuth-Based Materials

Bismuth-based materials have been widely explored as visible-light-responsive photocatalysts for environmental remediation owing to their favorable electronic structures, strong visible-light absorption, and long carrier lifetime. In particular, Bi-based oxides, oxyhalides, and chalcogenides have been extensively investigated for applications such as organic pollutant degradation, heavy-metal removal, and air and water purification. These studies establish a well-developed foundation for understanding the photo-induced behavior of Bi-based systems and provide an important reference framework for assessing performance improvements in emerging PEC and photo-driven technologies [94]. Adepu et al. reported a porous titanosilicate/BiVO_4_ (TS/BiVO_4_) heterostructured photocatalyst for the degradation of Rhodamine B under sunlight irradiation.

Among the synthesized composites, BVTS-1 (TS:BiVO_4_ = 2:1) exhibited the highest photocatalytic activity, achieving nearly complete RhB degradation within 60 min, whereas pristine titanosilicate and BiVO_4_ showed significantly lower performance. The enhanced performance was attributed to effective heterojunction formation, which improved visible-light absorption and facilitated excellent separation and transfer of photogenerated carriers. Band-structure analysis and mechanistic studies indicated that photogenerated electrons promoted the formation of superoxide radicals (^•^O_2_^−^), which subsequently generated hydroxyl radicals (^•^OH); together with photogenerated h^+^, these ROS played a dominant role in RhB degradation, resulting in superior photocatalytic performance under sunlight irradiation [95].

**Figure 5:**
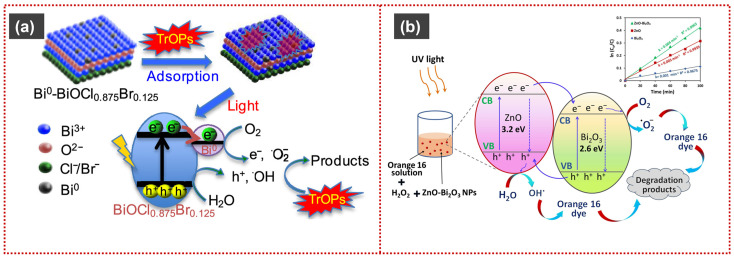
(**a**) Schematic illustration of TroPs degradation by Bi^0^-doped BiOCl_0.875_Br_0.125_. Reproduced from [97] with permission from the American Chemical Society. © 2018 Author(s). (**b**) Representation of Orange 16 Reactive Dye degradation mechanism. Reproduced from [98]. © 2023 Author(s). Licensed under CC BY 4.0.

Ke et al. reported a Bi_2_O_3_/Bi_2_S_3_/MoS_2_ n–p heterojunction photocatalyst with significantly enhanced photocatalytic activity under simulated solar irradiation. The heterojunction exhibited a substantially higher photocurrent response and lower charge-transfer resistance than the individual components, indicating improved charge separation and transport. It delivered an initial O_2_ evolution rate of 529.1 μmol h^−1^ g^−1^, approximately 1.5 and 12.5 times higher than those of pure Bi_2_O_3_ and MoS_2_, respectively, and achieved ~90% degradation of methylene blue. Electron paramagnetic resonance (EPR) analysis confirmed that ^•^O_2_^−^ and ^•^OH radicals were the primary reactive species responsible for the faster degradation kinetics [96]. Dandapat et al. reported the solar photocatalytic degradation of trace organic pollutants in water using Bi(0)-doped bismuth oxyhalide thin films. Among the investigated samples, 3% Bi(0)-doped BiOCl_0_._875_Br_0_._125_ exhibited the highest photocatalytic activity toward most tested pollutants, including sulfamethoxazole (SMX), carbamazepine (CBZ), venlafaxine (VLX), and bezafibrate (BZF), outperforming undoped bismuth oxyhalide films and TiO_2_ under solar irradiation. The enhanced performance was attributed to band-gap narrowing, improved visible-light absorption, and a highly ordered and compact morphology. Surface charge analysis excluded electrostatic interactions, while ion-exchange–driven adsorption was identified as the dominant mechanism, particularly for the high degradation rate of bezafibrate. The degradation process was proposed to proceed via pollutant adsorption followed by photocatalytic oxidative decomposition under solar illumination, as shown in Figure 5a [97].

Liu et al. reported exfoliated Bi_2_Se_3_ nanosheets for the degradation of MO and the reduction of hexavalent chromium [Cr(VI)]. Under visible-light irradiation, the Bi_2_Se_3_ nanosheets achieved 81.2% MO degradation within 240 min, with a pseudo-first-order rate constant of 0.0038 min^−1^. In comparison, Cr(VI) reduction reached 88.4% within 60 min with a rate constant of 0.007 min^−1^. The enhanced photocatalytic performance was attributed to its large surface area, efficient light absorption, and effective generation of e-h pairs. Mechanistic analysis indicated that ROS, particularly ^•^OH and ^•^O_2_^−^ radicals, played a key role in MO degradation. In contrast, photogenerated electrons were responsible for the reduction of Cr(VI) to the less toxic Cr(III) species [25]. The ZnO–Bi_2_O_3_ heterostructured photocatalyst exhibits enhanced photocatalytic degradation of Reactive Orange 16 under UV irradiation compared with pristine ZnO and Bi_2_O_3_. The heterojunction achieves approximately 48.5% dye removal in the presence of H_2_O_2_. Kinetic analysis follows pseudo-first-order behavior, with the highest rate constant (0.008 min^−1^) obtained for the ZnO–Bi_2_O_3_/UV/H_2_O_2_ system; the degradation mechanism is illustrated in Figure 5b. Mechanistic studies identify ^•^OH and ^•^O_2_^−^ radicals as the primary reactive species responsible for dye degradation [98].

Zhou et al. reported the photocatalytic degradation of tetracycline using bismuth silver oxide (BSO) perovskite nanocatalysts synthesized via a co-deposition method. Under ultraviolet irradiation, BSO achieved a maximum tetracycline degradation of 95.79% within 80 min, following pseudo-first-order kinetics with a rate constant of 0.0361 min^−1^. The photocatalytic activity was strongly influenced by catalyst dosage, initial pollutant concentration, pH, and Ag:Bi molar ratio, with optimal conditions of 60 mg L^−1^ tetracycline concentration, 2.0 g L^−1^ catalyst dosage, pH 8, and Ag:Bi = 1:1. Reusability tests showed that the degradation efficacy remained above 80% after four cycles, indicating good stability. Structural analyses revealed that BSO gradually transformed into Bi_2_O_2_CO_3_ during repeated photocatalytic reactions, while retaining high catalytic activity and sustaining tetracycline degradation [99].

Overall, these photocatalytic studies establish the strong visible-light responsiveness and broad applicability of Bi-based materials. However, intrinsic limitations, such as charge recombination, incomplete mineralization, and stability concerns, provide strong motivation for developing PEC systems, in which external bias and electrode engineering can further enhance charge utilization and reaction selectivity. A summary of PC is provided in Table 3.

## 5. Performance-Enhancing Modifications

From a synthesis-driven design perspective, the intrinsic properties of Bi-based semiconductors can be further optimized through post-synthetic modifications. These strategies directly enhance carrier lifetime, defect chemistry, and interfacial charge transfer. Bismuth-based photocatalysts (BBPs) have gained considerable attention for visible-light-driven environmental remediation. However, their practical application remains limited by low charge-carrier mobility, rapid e-h recombination, suboptimal band-gap positions, and limited visible-light absorption [100]. These intrinsic drawbacks collectively reduce the overall photocatalytic performance of BBPs for the treatment of organic pollutants and emerging contaminants in water treatment systems. Recent advances in performance-enhancing modification strategies, including elemental doping, defect engineering, and heterojunction formation, have improved light harvesting, enhanced interfacial kinetics, and increased stability, thereby enhancing the environmental remediation potential of Bi-based semiconductors.

### 5.1. Heterojunction Engineering

From a PEC-performance perspective, heterojunction engineering is a highly effective strategy. It overcomes key limitations of single-component Bi-based semiconductors, including suboptimal band gaps, higher recombination, and limited redox potentials [101,102]. An effective photocatalyst requires strong solar-light absorption and effective charge separation, with band edges well aligned to the target redox reactions [103].

**Figure 6:**
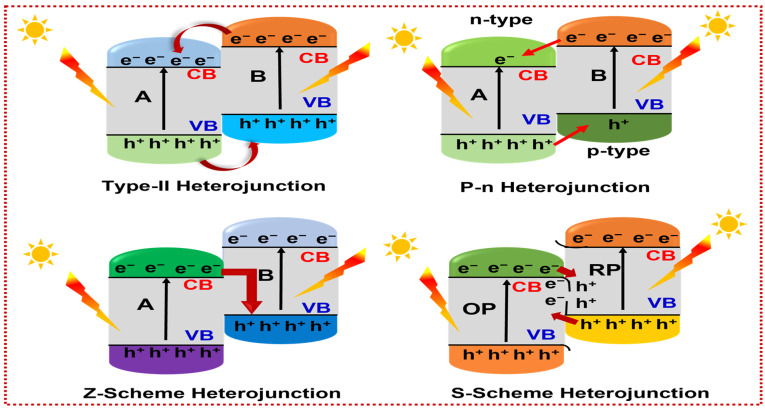
Schematic illustration of charge transfer and separation mechanisms in different semiconductor heterojunction photocatalysts under light irradiation.

Several key parameters dictate the performance of a heterojunction. Intimate interfacial contact between the constituent semiconductors is a prerequisite for effective charge separation and the establishment of a built-in electric field at the interface. Equally important is the relative alignment of the conduction and V_B_, which determines the direction and driving force for photogenerated e-h transfer and is strongly dependent on the heterojunction type. The magnitude of the internal electric field and the interfacial potential barrier are further governed by intrinsic semiconductor properties, including carrier type (n- or p-type), work function, and Fermi level alignment [104]. In addition to electronic factors, the interfacial geometry and orientation between the coupled components significantly influence charge-transport kinetics. Heterojunctions with different spatial configurations can exhibit distinct photocatalytic performances due to variations in contact area and charge-transfer pathways, as shown in Figure 6. In particular, configurations that maximize face-to-face contact have been shown to promote more effective interfacial carrier migration, thereby delivering superior catalytic activity [105].

Previous studies have highlighted that different heterojunction architectures can exhibit fundamentally distinct charge-transfer behaviors, and misinterpretation of these mechanisms may lead to incorrect conclusions regarding photocatalytic performance. According to the heterojunction classification framework proposed by Han et al., semiconductor heterojunctions can be categorized based on band alignment, Fermi-level equilibration, internal electric-field formation, and charge-transfer pathways. In Type-II heterojunctions, staggered band alignment drives photogenerated electrons and h^+^ to migrate into different semiconductors, enabling spatial charge separation but often at the expense of weakened redox capability. In contrast, p–n heterojunctions are formed by coupling p-type and n-type semiconductors, where Fermi-level equilibration induces band bending and a built-in electric field that directs carrier migration and suppresses recombination. In Z-scheme heterojunctions, low-energy electrons and h^+^ recombine at the interface, whereas high-energy electrons and h^+^ are retained in the respective semiconductors, thereby preserving strong redox ability. In contrast, S-scheme heterojunctions arise from interfacial band bending and internal electric-field effects induced by Fermi-level equilibration between semiconductors with different work functions, which selectively eliminate low-energy charge carriers and maintain spatially separated, high-energy electrons and h^+^ in reduction and oxidation photocatalysts, respectively [106,107,108].

#### 5.1.1. Type-II Heterojunction Systems

Type-II heterojunctions form between semiconductors with staggered band structures, where the C_B_ and V_B_ of one semiconductor lie at higher energies than those of the other. Electrons transfer to the lower C_B_, while h^+^ migrate to the higher VB. Interfacial contact induces Fermi-level equilibration and band bending, and the resulting internal electric field further extends carrier lifetime [102,109].

Zeng et al. developed a WO_3_/BiVO_4_ heterostructured photoanode in which nanoporous BiVO_4_ is uniformly coated on quasi-vertically aligned WO_3_ nanoplates, thereby creating a large area heterointerface. The WO_3_/BiVO_4_ electrode delivered a 2.83 mA cm^−2^ photocurrent density at 1.23 V vs. RHE. Mo doping further enhanced the photocurrent to 3.78 mA cm^−2^, and Co–Pi cocatalyst loading increased it to 5.38 mA cm^−2^. Electrochemical impedance spectroscopy (EIS) revealed a reduction in charge-transfer resistance after heterojunction formation and Mo incorporation, indicating faster interfacial charge transport. In PEC mode, the WO_3_/Mo-BiVO_4_ photoanode removed 85.6% tetracycline hydrochloride (k = 0.683 h^−1^) and efficiently degraded phenol and Congo red. The improvement was attributed to directional carrier separation in the quasi-type-II junction and enhanced h^+^ driven oxidation at the BiVO_4_ surface [110].

**Figure 7:**
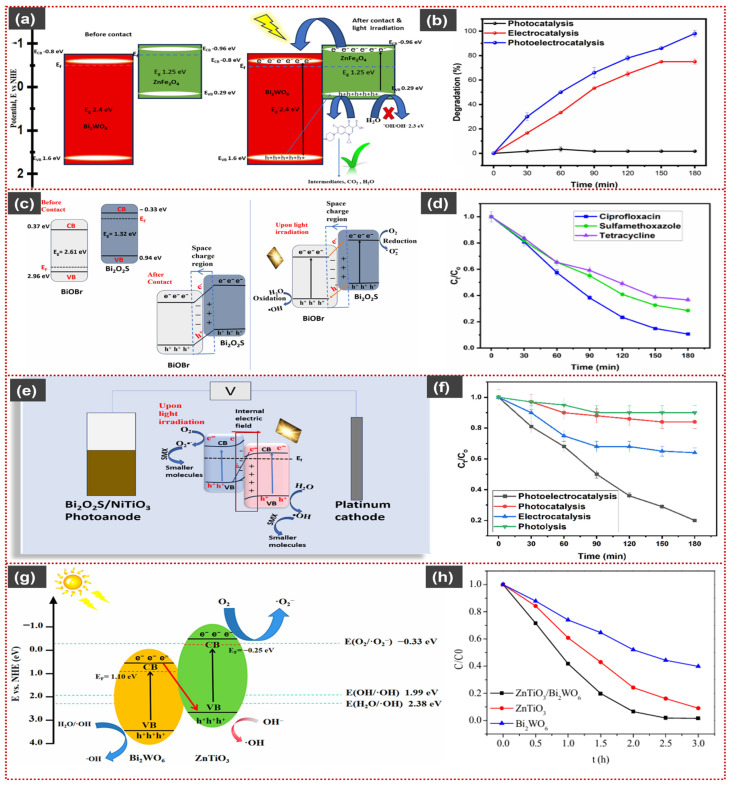
Representative heterojunction architectures and PEC performances of Bi-based systems: (**a**) schematic representation of charge transfer at Bi_2_WO_6_/ZnFe_2_O_4_ and (**b**) degradation of ciprofloxacin at Bi_2_WO_6_/ZnFe_2_O_4_. Reproduced with permission from [111], *Journal of Solid State Electrochemistry*, 2025, © the Author(s), licensed under CC BY 4.0. (**c**) Illustration of proposed heterojunction formation and degradation mechanism by Bi_2_O_2_S/BiOBr, (**d**) corresponding degradation curves of different analytes. Reproduced with permission from [115], *RSC Advances*, 2025, © the Author(s), licensed under CC BY 3.0. (**e**) Schematic charge transfer and degradation mechanism in Bi_2_O_2_S/NiTiO_3_, (**f**) PEC degradation of Sulfamethoxazole by Bi_2_O_2_S/NiTiO_3_. Reproduced with permission from [119], *ACS Applied Materials & Interfaces*, 2024, © the Author(s), licensed under CC BY 4.0. (**g**) S-scheme heterojunction formation and degradation mechanism by ZnTiO_3_ Nanosheets/Bi_2_WO_6_ and (**h**) corresponding degradation curves. Reproduced with permission from [124], *Molecules*, 2023, © the Author(s), licensed under CC BY 4.0.

Sebokolodi et al. demonstrated remarkable PEC degradation of ciprofloxacin over a Bi_2_WO_6_/ZnFe_2_O_4_ heterojunction photoanode, achieving ~98% pollutant removal within 180 min under visible light and low bias (Figure 7a,b). The process follows pseudo-first-order kinetics with a high rate constant of 1.689 × 10^−2^ min^−1^, significantly outperforming the photocatalytic and electrocatalytic routes alone. TOC analysis revealed 58.9% mineralization, confirming substantial breakdown beyond molecular transformation. Ultra-Performance Liquid Chromatography–Mass Spectrometry (UPLC–MS) analysis revealed progressive disappearance of the parent ciprofloxacin peak (*m*/*z* 332.14) and the formation of several lower-molecular-weight intermediates (*m*/*z* 302.19, 274.27, 226.95, 149.02, and 124.08), indicating stepwise degradation via decarboxylation, ring cleavage, and molecular fragmentation, leading to less complex and less toxic species prior to mineralization. Reactive-species trapping experiments identified photogenerated h^+^ as the dominant oxidizing species, with ^•^OH and ^•^O_2_^−^ playing minor roles [111].

Fan et al. reported that the BiVO_4_/BiOI photoanode shows a hierarchical nanoparticle–nanosheet architecture with intimate interfacial contact. The optimized BVOI-300 exhibits a high photocurrent (~3.4 mA cm^−2^ at 2 V vs. Ag/AgCl) and a low charge-transfer resistance (~59 Ω under illumination). It achieved 82% PEC degradation of β-naphthol within 8 h, with a pseudo-first-order rate constant of 3.64 × 10^−3^ min^−1^. Radical-trapping and electron spin resonance (ESR) spectroscopy identify ^•^OH, ^•^O_2_^−^, and h^+^ as the dominant reactive species, consistent with directional charge migration in the type-II junction [112]. In the PEC treatment of coal gasification wastewater, the TOC was reduced from 94.44 to 54.40 mg L^−1^, corresponding to approximaately 42.4% mineralization. Gas Chromatography–Mass Spectrometry (GC–MS) results showed substantial degradation of aromatic and nitrogen-containing compounds (e.g., p-xylene), accompanied by the formation of simpler alkane and ester intermediates. This suggests that the PEC process primarily converts refractory organics into less-toxic transformation products rather than achieving full mineralization [112]. Collectively, these results demonstrate that type-II heterojunctions improve carrier separation and photocurrent response, although partial loss of redox potential limits their ultimate PEC performance [105].

#### 5.1.2. p–n Heterojunctions

Conventional p–n heterojunctions are formed at the interface between an n-type and a p-type semiconductor. Upon contact, electrons diffuse from the n-type to the p-type region, while h^+^ migrate in the opposite direction, creating a depletion zone and a built-in induced electric field (IEF). Under illumination, this field drives photogenerated electrons toward the n-type side and h^+^ toward the p-type side, thereby suppressing recombination. This mechanism is advantageous for photocatalytic pollutant degradation and water splitting. However the redox potentials of the separated carriers are often lower than in Z-scheme or S-scheme systems, even when the band structures are correctly aligned, requiring stronger oxidative or reductive capabilities for optimal performance [109].

Chen et al. constructed a p–n heterojunction photoanode by depositing BiOI nanosheets onto highly ordered TiO_2_ nanotube arrays (BiOI-TNTAs). SEM images showed vertically aligned TiO_2_ nanotubes (~90–110 nm) that remained intact after BiOI nanosheet deposition, with BiOI uniformly covering the tube walls. The BiOI-TNTAs photoanode achieves ~0.147 mA at 1.2 V vs. Ag/AgCl, approximately 2.8 times that of bare TNTAs. EIS indicates a reduced charge-transfer resistance (Rp ≈ 343.32 Ω) and an extended electron lifetime (τ_el_ ≈ 3.24 ms). Ibuprofen is completely removed within 120 min with a rate constant k = 3.21 × 10^−2^ min^−1^, and 55.18% TOC removal. Based on band alignment and reported ESR evidence, h^+^ in BiOI promotes ^•^OH formation, which drives ibuprofen oxidation [113]. Mafa et al. reported that the g-C_3_N_4_/BiOI/EG composite forms a hierarchical flower-like BiOI/slate-like g-C_3_N_4_ interface with intimate contact. This architecture achieves a higher photocurrent density and lower charge-transfer resistance, enabling the removal of 88% sulfamethoxazole within 180 min [114].

Jayeola et al. reported a 2D/1D BiOBr/Bi_2_O_2_S p–n heterojunction photoanode, in which BiOBr nanosheets were in situ grown on Bi_2_O_2_S nanorods to form an interracially coupled architecture that suppresses bulk and surface recombination. Owing to the synergistic heterojunction effect, the optimized BiOBr/20% Bi_2_O_2_S electrode achieved 88% PEC degradation of ciprofloxacin within 180 min under visible-light irradiation (Figure 7c,d) at a low current density of 5 mA cm^−2^, significantly outperforming pristine BiOBr and Bi_2_O_2_S. The degradation followed pseudo-first-order kinetics with a rate constant of 0.0127 min^−1^, markedly higher than those for PC, electrocatalysis, or photolysis alone. TOC analysis confirmed ~60% mineralization, indicating substantial breakdown of the antibiotic beyond molecular transformation. UPLC–MS analysis showed the parent ciprofloxacin peak (*m*/*z* 332.14) gradually disappeared, accompanied by the formation of several lower-molecular-weight intermediates (*m*/*z* 302, 274, 202, 158, 124, 326, and 306), indicating stepwise degradation involving hydroxylation, decarboxylation, piperazine-ring cleavage, and aromatic ring opening. These unstable intermediates were further oxidized into smaller fragments, consistent with the observed TOC reduction and progressive mineralization. Reactive-species-trapping experiments revealed ^•^OH radicals as the dominant oxidative species, with ^•^O_2_^−^ and photogenerated h^+^ playing secondary roles, consistent with charge redistribution and space-charge-region formation in the p–n heterojunction, which suppresses recombination and enhances PEC oxidation efficiency [115].

Qin et al. reported that plasmonic Bi/Bi_2_O_3_/TiO_2_ nanotube photoanodes exhibit a well-defined hierarchical architecture. Bi nanoparticles and a thin Bi_2_O_3_ layer are integrated onto ordered TiO_2_ nanotubes. The p–n Bi_2_O_3_/TiO_2_ junction and plasmonic Bi improve visible-light absorption and boost photocurrent relative to pristine TiO_2_ and Bi_2_O_3_. Oxytetracycline removal reached 46.3% within 120 min (k = 5.04 × 10^−3^ min^−1^). In co-contaminant systems, oxytetracycline degradation reached 76.1%, and Cu^2+^ reduction reached 96.5% after 5 h. High-Performance Liquid Chromatography–Mass Spectrometry (HPLC–MS) analysis detected multiple oxytetracycline transformation products with mass-to-charge (*m*/*z*) ratios of 477, 447, 433, 362, 279, and 227, attributable to successive hydroxylation, demethylation, decarbonylation, and structural cleavage reactions. These identified intermediates are proposed to undergo further oxidation to smaller inorganic products such as CO_2_ and H_2_O. Trapping studies indicate h^+^ and ^•^OH as the primary oxidative species [116]. Although this strategy can effectively inhibit the recombination of photoinduced electrons with h^+^, the redox ability of the photocatalytic system is weakened [117].

#### 5.1.3. Z-Scheme Heterojunction Systems

Z-scheme photocatalytic systems have been developed to address the loss of redox power in type-II heterojunctions. In a Z-scheme configuration, low-energy electrons in the C_B_ of the oxidation photocatalyst (OP) recombine with low-energy h^+^ in the V_B_ of the reduction photocatalyst (RP). High-energy electrons remain in the C_B_ of the RP, while high-energy h^+^ stay in the VB of the OP. These carriers retain good reduction and oxidation abilities, preserving the overall redox strength of the system [109].

Tan et al. fabricated a Z-scheme BiVO_4_/NH_2_-MIL-125(Ti) (BiVO_4_/NM125) photoanode via a one-step solvothermal route. SEM images showed that decahedral NM125 particles are uniformly anchored on compact BiVO_4_ films (~1 µm thick), forming intimate interfacial contact without disrupting the BiVO_4_ framework. The BiVO_4_/NM125 photoanode exhibited a higher photocurrent than pristine BiVO_4_ and NM125. Among the samples, BiVO_4_/1NM125 exhibited the strongest transient response and an incident photon to current efficiency of ~2.6% at 420 nm. EIS analysis reveals a markedly reduced charge-transfer resistance (Rct ≈ 2.93 kΩ), indicating improved interfacial charge transport. In PEC phenol degradation, the optimized photoanode achieved 96.7% removal within 150 min at a 2.0 V bias. For chemical oxygen demand (COD), approximately 70% was measured, indicating substantial phenol oxidation. HPLC results revealed p-benzoquinone as a transient intermediate that was further degraded under prolonged PEC treatment, suggesting stepwise oxidation toward mineralization in CO_2_ and H_2_O. The reaction followed pseudo-first-order kinetics with a rate constant of 0.0222 min^−1^. Radical trapping experiments identify h^+^, ^•^OH, and ^•^O_2_^−^ as active species, with ^•^O_2_^−^ playing the dominant role. These results are consistent with a Z-scheme charge-transfer pathway that preserves strong redox capability [118].

Jayeola et al. reported an in situ-grown Z-scheme Bi_2_O_2_S/25% NiTiO_3_ heterojunction photoanode, in which Bi_2_O_2_S nanoparticles were uniformly anchored to plate-like NiTiO_3_, forming an intimate, highly intertwined interface. This architecture promotes efficient interfacial charge separation and suppresses carrier recombination via an interfacial S–O bond. Under visible-light PEC conditions, it achieved about 80% degradation of sulfamethoxazole (Figure 7e,f) within 180 min at a low current density of 5 mA cm^−2^, following pseudo-first-order kinetics with a rate constant of 0.0088 min^−1^. TOC analysis revealed ~45.5% mineralization, indicating partial but substantial conversion of the pollutant into smaller inorganic species. Radical-scavenging experiments identified ^•^OH as the dominant oxidative species, with ^•^O_2_^−^ playing a secondary role, confirming a Z-scheme-driven degradation mechanism [119].

Feng et al. reported a dual Z-scheme Bi_2_S_3_/Bi_2_O_3_/WO_3_ ternary photoanode fabricated by sequential deposition and in situ anion exchange. Bi_2_S_3_ particles uniformly decorate Bi_2_O_3_-modified, needle-like WO_3_ frameworks, forming a hierarchical architecture. The multicomponent structure enhances visible-light absorption and suppresses charge recombination. The dual Z-scheme pathway promotes efficient interfacial charge transfer and photo–electro synergy. The Bi_2_S_3_/Bi_2_O_3_/WO_3_ film achieves 84.2% RhB degradation within 180 min (k = 7.37 × 10^−3^ min^−1^). It also removes 61.1% diclofenac (k = 2.29 × 10^−3^ min^−1^) under visible light at +1.0 V. Reactive-species analysis identified ^•^OH and ^•^O_2_^−^ as the dominant oxidative species in the dual Z-scheme PEC system [120].

Z-scheme heterojunctions effectively enhance charge-carrier separation, extend the spectral range of visible light utilization, and retain the high redox potentials of the constituent semiconductors. However, charge recombination is not eliminated, as electrons residing in the relatively higher C_B_ and h^+^ in the lower V_B_ may still undergo undesired back-transfer, particularly under prolonged illumination or high carrier densities [105].

#### 5.1.4. S-Scheme Heterojunction Systems

S-scheme heterojunctions represent a more recent conceptual advancement that integrates the benefits of both type-II and Z-scheme configurations while minimizing their drawbacks. In an S-scheme system, an RP with a higher C_B_ and Fermi level is coupled with an OP with a lower Fermi level. Upon contact, electron transfer from the OP to the RP and h^+^ transfer in the opposite direction establish an internal electric field at the interface and induce band bending. Under illumination, low-energy carriers recombine at the interface. High-energy electrons in the RP and h^+^ in the OP remain spatially separated. This preserves better redox capability without external redox mediators [121].

Jayeola et al. reported a CeO_2_/Bi_2_O_2_S S-scheme heterojunction, synthesized via an in situ hydrothermal method. SEM images revealed well-dispersed Bi_2_O_2_S with increased surface area after CeO_2_ coupling. EIS showed a reduction in charge-transfer resistance from 205 Ω (Bi_2_O_2_S) to 112 Ω (CeO_2_/Bi_2_O_2_S). Photocurrent measurements confirm suppressed recombination, supporting the effectiveness of the S-scheme charge-transfer. At near-neutral pH (6.7), degradation increases to 87%. The photoanode achieved 72% TOC removal of sulfamethoxazole in synthetic wastewater after 180 min and 54% removal in real wastewater. UPLC–MS analysis identified several sulfamethoxazole transformation products, including N-((hydroxyamino)methyl)benzenesulfonamide (*m*/*z* 203.3), benzenesulfinamide (*m*/*z* 141.9), hydrosulfinylbenzene (*m*/*z* 124.08), and butan-1,4-dioic acid (*m*/*z* 118.09), indicating stepwise degradation involving ring opening and functional group loss [103,122]. Sipuka et al. synthesized a ZnO/Bi_3_TaO_7_ (ZBTO) S-scheme heterojunction photoanode by a hydrothermal method. SEM images revealed spherical Bi_3_TaO_7_ nanoparticles intimately integrated with rod-like ZnO structures. The ZBTO photoanode delivers a photocurrent density of 0.036 mA cm^−2^, higher than that of Bi_3_TaO_7_ and ZnO. EIS reveals a reduced charge-transfer resistance (Rct ≈ 89 Ω). Under optimal conditions (pH 7, 5 mA cm^−2^), the photoanode achieved 98% degradation of ciprofloxacin. The reaction followed pseudo-first-order kinetics with k = 2.23 × 10^−2^ min^−1^. TOC analysis revealed mineralization efficiencies of 76% and 55% for synthetic and real wastewater, respectively, indicating substantial mineralization. UPLC–MS identified several ciprofloxacin transformation products (*m*/*z* 306, 304, 263, 149, and 124), suggesting stepwise degradation via piperazine ring oxidation, decarboxylation, and molecular fragmentation, accompanied by reduced toxicity of the final intermediates. Radical scavenging identified ^•^OH and h^+^ as dominant species, consistent with S-scheme charge separation [123].

Zuo et al. reported a 2D/2D ZnTiO_3_ nanosheets/Bi_2_WO_6_ nanosheets S-scheme heterojunction photoanode, fabricated via a combined two-step calcination and hydrothermal method. SEM and TEM analyses revealed tightly interleaved large Bi_2_WO_6_ nanosheets and smaller ZnTiO_3_ nanosheets, forming abundant interfacial contacts that shorten charge-transfer pathways and suppress carrier recombination. Owing to the S-scheme charge-transfer mechanism and photoelectric coupling under a low applied bias (+0.5 V), the optimized ZnTiO_3_/Bi_2_WO_6_ electrode (ZnTiO_3_:Bi_2_WO_6_ = 1.5:1) achieved 93% PEC degradation of phenol within 180 min under visible light (Figure 7g,h), with a kinetic rate constant 3.6 times higher than pristine Bi_2_WO_6_. Electrochemical impedance and transient photocurrent analyses confirmed significantly reduced charge-transfer resistance (0.158 kΩ) and markedly enhanced photocurrent response. By-product analysis using GC–MS identified sequential degradation intermediates (*m*/*z* 94, 110, 108, and 142), corresponding to phenol → hydroquinone/catechol → p-benzoquinone → maleic acid, indicating stepwise ring oxidation and cleavage. Radical-trapping and ESR studies verified ^•^O_2_^−^ and h^+^ as the dominant reactive species, with ^•^OH acting as a secondary oxidant, consistent with efficient spatial charge separation in the S-scheme heterojunction [124].

Wu et al. reported that ultrafine Bi_2_Sn_2_O_7_ quantum dots (~6.6 nm) are uniformly anchored on vertically aligned TiO_2_ NTAs, forming an S-scheme heterojunction. The heterostructure exhibits an enhanced photocurrent density of 68.7 µA cm^−2^. Charge-transfer resistance is significantly reduced (Rct ≈ 26.4 Ω), indicating faster carrier separation. The photoanode achieved 90.3% PEC degradation of sulfamethazine within 120 min at 1.0 V, with a pseudo-first-order rate constant of 0.0189 min^−1^. The TOC measured was about 57.8%. Radical-quenching and ESR analyses identified h^+^, ^•^O_2_^−^ and ^1^O_2_ as the dominant reactive species [125]. Consequently, S-scheme heterojunctions are often considered the most advanced strategy for minimizing recombination and maximizing redox strength in Bi-based PEC. According to this study, Type-II heterojunctions suffer from weakened redox capability despite improved charge separation, while p–n heterojunctions remain highly dependent on junction quality. Z-scheme heterojunctions retain strong redox capability but still experience interfacial recombination. In contrast, the S-scheme heterojunction is considered the most advanced architecture, as it simultaneously enhances carrier separation and maintains strong redox potential, thereby overcoming the intrinsic limitations of the other heterojunction types [105].

### 5.2. Doping

Elemental doping is one of the most effective approaches to tailor the optoelectronic properties and environmental stability of Bi-based semiconductors by modulating their band structure, carrier dynamics, and surface chemistry [126]. Doping strategies are generally classified as metallic, non-metallic, and self-doping [127], each influencing the electronic structure in distinct but complementary ways. Metallic dopants can modify crystallinity, introduce lattice defects, extend light absorption into the visible region, and improve the separation efficiency of photogenerated e–h pairs [101,128].

Tsay et al. reported the hydrothermal synthesis of pristine BiVO_4_ and its W- and Mo-doped derivatives (BiVO_4_:W and BiVO_4_:Mo), which were evaluated for Rhodamine B (RhB) degradation under visible light. Incorporation of W and Mo refined the particle morphology, reduced particle size, and increased the specific surface area. BiVO_4_:Mo exhibited the highest surface area (8.21 m^2^ g^−1^). XRD indicated that all samples retained the monoclinic BiVO_4_ structure, whereas slight shifts in diffraction peaks indicated successful dopant incorporation. The average particle size decreased from 164 nm for pristine BiVO_4_ to 137 nm for BiVO_4_:W and 135 nm for BiVO_4_:Mo, which correlated with improved photocatalytic activity. BiVO_4_:Mo exhibited the highest RhB degradation efficiency (86.8%), followed by BiVO_4_:W (74.4%) and undoped BiVO_4_ (61.8%). Fluorescence spectroscopy further validated more efficient e–h separation in the doped samples, demonstrating that W and Mo doping substantially enhances the visible-light photocatalytic performance of BiVO_4_ for pollutant degradation [129].

Non-metallic doping in BBPs introduces localized energy levels between the C_B_ and V_B_, thereby enhancing visible-light absorption and improving light utilization efficiency [130]. Such dopants also facilitate charge transfer within the semiconductor, promoting effective e–h separation. Evidence suggests that self-doping with bromide ions reduces the band gap, enhances adsorption capacity, accelerates carrier transport, and improves e-h separation in the material [131]. Wu et al. reported Boron-doped BiOBr nanosheets for the photocatalytic inactivation of Escherichia coli K-12. The introduction of B centres created additional electron-acceptor sites within the BiOBr lattice, thereby enhancing carrier migration and improving the disinfection performance [132].

**Figure 8:**
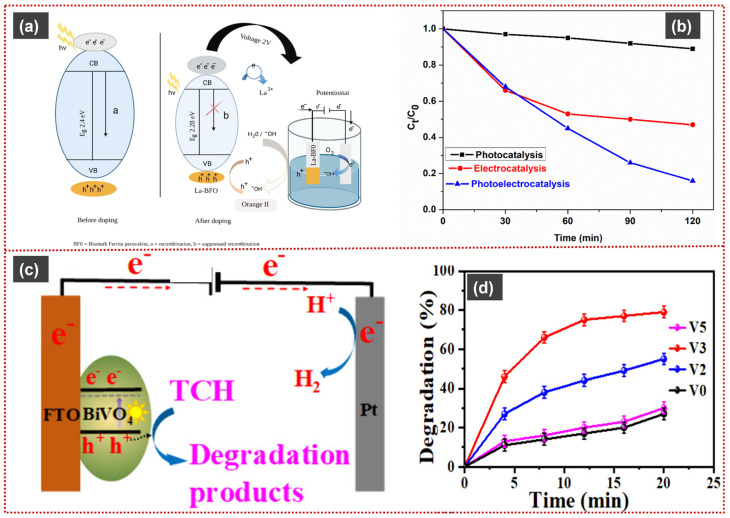
(**a**) Schematic illustration of dopant-induced interfacial charge transfer in La^3+^-doped BiFeO_3_; (**b**) corresponding PEC degradation of Orange II. Reproduced with permission from [45], *Catalysts*, 2021, © the Author(s), licensed under CC BY 4.0. (**c**) Schematic illustration of the proposed PEC degradation mechanism of TCH. (**d**) Degradation curves of TCH. Reprinted with permission from [139], copyright © 2024 American Chemical Society.

Nkwachukwu et al. reported La^3+^-doped BiFeO_3_ (La-BFO) photoanodes preserve the perovskite morphology of pristine BFO, while exhibiting markedly improved PEC performance. The optimized 10% La-BFO electrode exhibited approximately 3x higher photocurrent density (0.118 mA cm^−2^) and much lower charge-transfer resistance (~406 Ω) than undoped BFO, indicating faster carrier transport and reduced bulk/interface losses. Consequently, the photoanode achieves 84.2% PEC degradation of Orange II, as shown in Figure 8a,b, within 120 min at 2 V, with a rate constant of 1.54 × 10^−2^ min^−1^. Radical-scavenging and band-edge analyses identify photogenerated h^+^ and ^•^OH as the primary oxidative species, while ^•^O_2_^−^ plays a negligible role. These findings demonstrate that La^3+^ doping effectively tailors the electronic structure of BiFeO_3_, enhancing ROS-driven oxidation for pollutant degradation [45].

Although doping enhances PEC activity, long-term stability remains a concern due to thermodynamic instability, lattice distortion, and dopant segregation, which may ultimately lead to dopant loss under extended operation [133]. Recent studies have demonstrated that elemental doping, while beneficial for enhancing catalytic activity, can introduce intrinsic trade-offs in stability due to dopant loss under operating conditions. For example, in Mo-modified transition-metal oxyhydroxide catalysts, operando Raman spectroscopy, X-ray photoelectron spectroscopy (XPS), and inductively coupled plasma–mass spectrometry (ICP-MS) analyses revealed that Mo–O_3X_ species progressively leached from the host lattice during electrochemical operation. This dopant leaching leads to a measurable decrease in surface Mo concentration and is accompanied by the formation of O_v_ and local electronic reconstruction. Although such dynamic dopant removal can transiently enhance catalytic activity, excessive dopant loss ultimately alters the catalyst composition and may compromise long-term durability [134]. These findings highlight that dopant incorporation does not necessarily guarantee dopant retention, underscoring the importance of balancing activity gains with stability considerations when designing doped photo(electro)catalysts for sustained operation.

### 5.3. Defect Engineering

Defect engineering, especially the controlled introduction of O_v_, is a key strategy for improving BBPs’ performance. O_v_ acts as a shallow electron donor and introduces defect states within the band gap. These effects increase charge density and enhance conductivity [135,136]. Xin et al. reported that in BiVO_4_, the presence of O_v_ narrowed the band gap, broadened visible-light absorption, and increased photocurrent density by modulating the band structure and promoting rapid e–h separation [137]. O_v_ facilitates the generation of ROS, including ^•^O_2_^−^ and ^•^OH radicals, which play central roles in photocatalytic pollutant degradation.

Ma et al. reported that FeOOH coating effectively stabilized surface O_v_ in BiVO_4_ during PEC operation, preserving defect structures and improving long-term performance. O_v_ engineering markedly enhanced activity in BiVO_4_ and BiOBr systems. The BiVO_4_ photocurrent increased from 0.39 to 0.54 mA cm^−2^ with O_v_ introduction. When combined with an FeOOH cocatalyst, the photocurrent further increased to 1.18 mA cm^−2^, achieving 85% RhB PEC degradation, underscoring the strong synergy between vacancy engineering and cocatalyst modification in facilitating pollutant oxidation [136]. Likewise, Pan et al. reported that protective shells such as NiFe-MOFs can maintain the integrity of oxygen-vacancy-rich surfaces under oxidative conditions, thereby sustaining high catalytic activity [135].

Sivasubramanian et al. reported B-doped oxygen-vacancy-rich Bi_2_Sn_2_O_7_ (B-BSO-O_V_) photoanodes form a uniform quantum-dot coating (~8.5 nm) on conductive Ni foam. This structure provides abundant defect sites and intimate interfacial contact for charge transport. Owing to the synergistic effects of boron doping and O_v_, the optimized electrode exhibited an improved photocurrent density (~18.9 µA cm^−2^). The electrode also exhibited a markedly lower charge-transfer resistance (~755 Ω) relative to pristine Bi_2_Sn_2_O. Consequently, complete degradation of sulfamethazine was achieved within 60 min at low bias (1.15 V). The process exhibits a high kinetic rate constant of 0.158 min^−1^, which is far higher than that of the defect-free counterpart. TOC analysis revealed approximately 68.5%, indicating substantial mineralization. UPLC–MS identified multiple transformation products, including sulfanilic acid, pyrimidine derivatives, and low-molecular-weight fragments (*m*/*z* 71–229), suggesting stepwise degradation via bond cleavage, oxidation, and ring opening, with final mineralization products inferred rather than directly measured. Reactive-species trapping and EPR analyses identify photogenerated h^+^ as the dominant oxidative species, with ^1^O_2_ as a secondary contributor, while ^•^O_2_^−^ and ^•^OH play minor roles [138].

Huang et al. reported bismuth- and oxygen–dual-vacancy-engineered BiVO_4_ photoanodes fabricated via co-sputtering of BiVO_4_ and V targets on FTO substrates, enabling precise regulation of defect concentrations. SEM images of the optimized photoanode revealed a dense, uniformly packed BiVO_4_ nanograined film with enhanced interparticle connectivity. The optimized photoanode exhibited the highest bismuth vacancy (~12%) and abundant O_v_, resulting in significantly enhanced carrier density. Under AM 1.5 illumination, the optimized electrode delivered a markedly improved photocurrent density of 1.9 mA cm^−2^ at 1.6 Vs RHE, which is ~11.9 times higher than that of vacancy-free BiVO_4_. EIS reveals a substantially reduced bulk charge-transfer resistance (~817 Ω), indicating efficient carrier transport. Consequently, the optimized photoanode achieves ~79% tetracycline hydrochloride degradation within 20 min under low bias (Figure 8c,d), following pseudo-first-order kinetics with a high-rate constant of 0.117 min^−1^. Radical trapping experiments demonstrated that photogenerated h^+^ are the dominant oxidative species, while ^•^OH and ^•^O_2_^−^ play minor roles. The synergistic introduction of bismuth and O_v_ effectively maintains the prolonged carrier lifetime, leading to superior PEC degradation performance [139].

O_v_ engineering is widely employed to enhance visible-light absorption, charge-carrier separation, and surface reaction kinetics in bismuth-based photo(electro)catalysts. However, excessive or poorly stabilized O_v_ may introduce deep trap states that accelerate nonradiative recombination and induce lattice distortion during prolonged operation. Under photo(electro)chemical conditions, high vacancy densities can also exacerbate photocorrosion and surface reconstruction, ultimately leading to gradual performance decay. These observations highlight that O_v_ concentration and stability must be carefully optimized, often in combination with defect passivation, heterojunction construction, or cocatalyst coupling, to balance activity enhancement with long-term structural and electrochemical stability [140,141,142].

Collectively, these findings underline the importance of oxygen-vacancy engineering in BBPs. Properly tuned O_v_ concentrations not only improve light absorption and charge transport but also promote ROS formation and provide abundant reaction sites. Defect-engineered Bi-based materials exhibit improved PEC and photocatalytic performance, as well as enhanced stability for environmental remediation when combined with suitable cocatalysts.

Building on the above discussions of heterojunction construction, elemental doping, and oxygen-vacancy engineering, it is clear that the synergistic regulation of electronic structure and interfacial charge-transfer processes governs the PEC performance of bismuth-based semiconductors. The integration of these modification strategies has enabled the development of a wide variety of high-performance Bi-based PEC systems for pollutant degradation. Owing to differences in semiconductor composition, electrode configuration, modification approach, operating conditions, and target contaminants, these systems exhibit diverse degradation behaviors and mechanistic characteristics. To facilitate a clear comparison, Table 4 summarizes representative bismuth-based PEC systems reported for the degradation of organic pollutants and emerging contaminants, providing a concise overview to guide the rational design of advanced Bi-based PEC electrodes.

## 6. Practical Considerations and Translation Challenges

### 6.1. Long-Term Stability, Durability, and Recyclability

Beyond initial photoactivity, long-term stability, durability, and recyclability are decisive factors governing the practical viability of bismuth-based photo(electro)catalytic systems. Under continuous PEC operation, photoelectrodes are exposed to prolonged light irradiation, external bias, highly reactive radical species, and complex aqueous environments, which can trigger photocorrosion, defect annihilation, interfacial degradation, and leaching of bismuth and halide species, ultimately compromising performance and material integrity. Consequently, high initial degradation efficiencies do not necessarily correlate with sustained long-term activity or operational robustness.

Most reported studies demonstrate apparent stability over a limited number of consecutive cycles; however, such short-term cycling tests often fail to capture slow degradation pathways that emerge under prolonged operation. Reported durability challenges in Bi-based PEC systems include phase transformation, instability or passivation of defect-derived active sites, structural instability of bismuth oxyhalides, and increased charge-transfer resistance at the semiconductor–electrolyte interface. These effects are further influenced by complex aqueous environments and prolonged operating conditions [156].

BiVO_4_, one of the most extensively studied Bi-based photoanodes, still suffers from performance limitations due to rapid recombination, sluggish surface water-oxidation kinetics, and photocorrosion/dissolution effects (including light-induced V^5+^ leaching) during extended PEC operation [157]. Studies reporting strategies to improve photostability indicate that surface modifications, cocatalyst integration, and protective layers can mitigate decay mechanisms by enhancing interfacial charge transfer and suppressing surface reconstruction or photocorrosion [158].

To address durability limitations, various material-level strategies have been developed for Bi-based photocatalysts. The construction of heterojunctions with built-in electric fields effectively suppresses recombination, thereby improving structural stability. Cocatalyst coupling further accelerates interfacial charge transfer and mitigates photocorrosion, while surface modification and protective shell architectures help preserve crystal integrity during repeated operation. In addition, improved recyclability and durability are commonly demonstrated through stable photocatalytic performance over multiple cycles, highlighting the effectiveness of these stabilization strategies [156].

Despite significant progress, standardized long-term evaluation protocols for Bi-based PEC systems remain limited. Most studies rely on short-term stability tests under idealized conditions, while extended continuous operation, quantitative leaching analysis, and systematic post-reaction structural characterization are not consistently reported. Establishing standardized assessment criteria is therefore essential for reliably evaluating the long-term durability, environmental safety, and practical applicability of bismuth-based PEC technologies [156].

### 6.2. Scalability and Techno-Economic Constraints of PEC Systems

Although bismuth-based PEC systems exhibit reduced recombination and improved controllability compared with particulate PC, their scalability is constrained by system-level and economic factors. A major limitation is the frequent need for an external bias to achieve meaningful photocurrent densities, which increases energy consumption and complicates reactor design. The applied bias, together with ohmic losses in the electrode and electrolyte, as well as interfacial carrier-transfer overpotentials, directly impacts overall energy efficiency and operating costs of PEC-based wastewater treatment processes [7,159].

From a manufacturing perspective, large-area fabrication of photoelectrodes with uniform thickness, strong substrate adhesion, and reproducible electronic properties remained challenging. While scalable deposition techniques such as spray pyrolysis, electrodeposition, and solution-based coating methods have been increasingly reported for environmental PEC applications, their techno-economic viability depends on precursor utilization efficiency, process throughput, and yield consistency. In addition, the reliance on conductive substrates (e.g., FTO, ITO, or Ti) and post-deposition thermal or chemical treatments can substantially increase material and processing costs.

Operational stability further influences economic feasibility. Performance decay associated with photocorrosion, interfacial delamination, fouling in complex water matrices, or degradation under sustained bias operation leads to increased maintenance requirements and shortened electrode lifetimes [160]. These stability-related challenges, widely reported in PEC wastewater treatment studies, collectively limit the cost competitiveness of PEC systems relative to established treatment technologies. This underscores the need to realistically assess energy input, material durability, and system complexity when evaluating the scalability of bismuth-based PEC remediation platforms.

## 7. Critical Assessment of Strategy Effectiveness

Recent analyses indicate that many PEC enhancement strategies fail because they improve short-term photocurrent without resolving the intrinsic instability of semiconductor photoanodes. In particular, materials whose self-oxidation or self-reduction potentials lie within the water redox window, such as representative Bi-based oxides and chalcogenides, remain thermodynamically susceptible to lattice degradation, regardless of improved light absorption. In many cases, band-gap narrowing, defect introduction, or heterojunction construction increases carrier generation. Still, it does not ensure efficient charge extraction, leading to hole accumulation at the semiconductor–electrolyte interface and accelerated photocorrosion. Surface passivation and cocatalyst loading can also be ineffective when interfacial band mismatch, poor electrical conductivity, or incomplete coverage introduce additional recombination pathways. Moreover, strategies that neglect electrolyte chemistry frequently fail, as pH and ionic composition strongly govern dissolution and leaching behavior in Bi-based PEC systems. Consequently, only integrated approaches that simultaneously address intrinsic stability, charge separation, surface reaction kinetics, and electrolyte compatibility have demonstrated sustained PEC performance [161].

Mechanistic analyses indicate that PEC enhancement strategies are effective only when they suppress the fundamental drivers of photoanode degradation. Effective approaches prevent the accumulation of photogenerated carriers at the semiconductor electrolyte interface by enabling efficient carrier extraction and surface reaction kinetics, thereby mitigating photocorrosion. Improvements based solely on band-gap narrowing, defect introduction, or heterojunction construction often fail if thermodynamic stability and interfacial charge transport are not addressed simultaneously. In addition, surface modification and cocatalyst loading are effective only when they maintain favorable band alignment and conductivity. At the same time, electrolyte chemistry plays a decisive role in controlling dissolution and leaching, particularly for Bi-based photoanodes. Consequently, Zhang et al. conclude that durable PEC performance arises from integrated designs that couple intrinsic material stability with interface and electrolyte engineering [161].

## 8. Environmental Applications of Bi-Based Photo(Electro)Catalysts

### 8.1. Performance for Model-Pollutant

From a practical application perspective, the structural and electronic advantages of Bi-based PEC translate into strong remediation performance. Bismuth-based semiconductors, including Bi_2_O_3_, Bi_2_S_3_, Bi_2_MO_6_ (M = Cr, Mo, W), BiVO_4_, BiOX (X = Cl, Br, I), metallic Bi, and Bi-based perovskites, are widely investigated in environmental remediation. BBPs efficiently degrade a wide range of pollutants under visible light, including pharmaceuticals, phthalates, tetracycline, RhB, and methylene blue. Excellent removal efficiency can be achieved through strategies such as doping, defect engineering, and heterojunction construction. Notably, the composite of metal BBPs and metal Bi-based Z- and S-scheme heterojunctions is considered particularly attractive for water purification, as these materials exhibit relatively high redox potential and enhanced charge-carrier separation [21,162].

#### 8.1.1. Degradation of Organic Pollutants in Water

Extensive studies demonstrate the high performance of BBPs in degrading organic dyes and model pollutants, including RhB, MB, MO, and phenol. One of the most prominent series is the Bi_2_WO_6_-based systems. Spray-pyrolyzed Bi_2_WO_6_ films showed a photocurrent density of 460 μA cm^−2^, whereas large-area photoelectrodes showed 94% PEC RhB degradation, which was much larger than the 23% removal achieved in pure PC mode. These findings demonstrate the feasibility of an engineered Bi_2_WO_6_ photoanode for efficient visible-light-driven degradation of dye [163]. The BiOX (X = Cl, Br, I) photocatalysts and their composites also exhibited outstanding activity for the removal of dyes in aqueous solution. In-depth studies highlight the critical contributions of defect engineering, construction of heterojunctions (such as BiOBr/BiOI, and BiOCl/g-C_3_N_4_), and carbonaceous supports in enabling efficient mineralization of dyes and phenolic pollutants under mild conditions [164]. Similarly, TiO_2_/Bi_2_O_3_ NP arrays achieved 100% RhB degradation under visible-light irradiation, highlighting the efficiency of Bi-based systems for dye degradation [143].

In addition to model dyes, pharmaceuticals, and other emerging organic contaminants (EOCs) have also been widely investigated. Multiple studies have shown that BiVO_4_, Bi_2_WO_6_, BiFeO_3_, Bi_2_MoO_6_, and Bi_2_S_3_-based catalysts are effective for degrading tetracyclines, sulfonamides, and fluoroquinolones. Many reports demonstrate 80–100% removal and substantial TOC reduction under visible-light irradiation, depending on the matrix and operating conditions; analgesics such as diclofenac, ibuprofen, β-lactams such as amoxicillin, and other pharmaceuticals and personal care products (PPCPs) are also removed [165,166]. Notably, 97.45% of amoxicillin is removed within 90 min under visible light by using BiVO_4_ thin-film photoelectrodes fabricated by electrophoretic deposition, demonstrating the promise of immobilized Bi-based coatings for antibiotic degradation [167]. Hydrothermally prepared BiVO_4_ nanoparticles also exhibited high photoactivity toward several tetracycline antibiotics, supporting the high affinity of BiVO_4_ for complex pharmaceutical pollutants [168].

The Ag–BiVO_4_/BiOI p–n heterojunction photoanode paired with the Ag–BiOI photocathode exhibited 92% PEC removal of 10 mg L^−1^ diclofenac sodium within 2 h at 1.0 V vs. Ag/AgCl under simulated sunlight. With this dual-photoelectrode arrangement, TOC removal increased from 41% (single photoanode) to ~63%, indicating a synergistic increase of the apparent rate constant [144]. Similarly, PEC advanced oxidation systems (PEC-AOSs) have been developed using WO_3_/BiVO_4_ photoanodes to degrade tetracycline, amoxicillin, and diclofenac; the WO_3_/BiVO_4_ junction enhances photocurrent response and leads to higher mineralization under low bias even in neutral electrolytes [169]. FeOOH/BiVO_4_ photoanodes also demonstrated that PEC oxidation coupled with peroxymonosulfate (PMS) activation can enhance the generation of **^•^**OH and SO_4_**^•−^** radicals, thereby likely deep degradation towards TCH as high as 88% [170]. The selective literature on antibiotic degradation supports that BBPs offer better removal efficiencies, good reusability, and broad applicability across different antibiotics. Heterojunctions between BiVO_4_, Bi_2_WO_6_, Bi_2_MoO_6_, and the various forms of BiOX commonly exceed 90% removal of tetracycline, sulfamethoxazole, ciprofloxacin, norfloxacin, and lomefloxacin, with substantial mineralization and minimal toxicity of intermediates in most cases [3].

#### 8.1.2. Disinfection of Algal/Bacterial Toxins

Under visible-light irradiation, Bi-based materials generate reactive species (^•^OH, ^•^O_2_^−^, h^+^) capable of inactivating microorganisms and degrading extracellular toxins, often achieving high inactivation or degradation efficiencies within 30–90 min, especially when coupled with cocatalysts or heterojunction partners [100]. More recently, Bi-based catalysts have been used to degrade microcystin-LR (MC-LR), a representative cyanobacterial toxin. Fe_2_O_3_/Bi_2_WO_6_ photocatalysts have been employed for MC-LR removal, in which V_B_-derived h^+^ and ^•^OH radicals played key roles in opening the cyclic peptide ring and promoting mineralization of the toxin. These outcomes highlight the potential of Bi-based systems for mitigating algal blooms and their associated health risks [171]. Rather and Lo et al. demonstrated effective PEC disinfection of Escherichia coli using a BiVO_4_-based g-C_3_N_4_/Ag/AgCl/BiVO_4_ (CAB-1) photoanode operated at 1.23 V vs. RHE under simulated solar irradiation in real municipal sewage. The Bi-based heterojunction enabled efficient charge separation and sustained generation of oxidative species, reducing E. coli to ~1000 CFU, which satisfies regulatory discharge limits for tertiary wastewater treatment [172].

Verma et al. demonstrated that quercetin-assisted Bi_2_S_3_ nanoparticles exhibit strong photocatalytic antibacterial and toxin-inactivation capability under visible-light irradiation. The Bi_2_S_3_ system effectively inactivated both Gram-negative (*E. coli*) and Gram-positive (*E. faecalis* and *B. subtilis*) bacteria via photoinduced ROS generation, resulting in membrane damage, leakage of intracellular components, and suppression of bacterial metabolic activity. Importantly, cytotoxicity and phytotoxicity assays confirmed minimal secondary toxicity, indicating the potential of Bi_2_S_3_-based photocatalysts for safe mitigation of bacterial toxins in water treatment systems. Quercetin-supported Bi_2_S_3_ nanoparticles are a versatile photocatalyst for the treatment of harmful bacteria and toxic cationic dyes in water [173].

#### 8.1.3. Heavy-Metal Ion Reduction

Bi-based composites have also been systematically investigated for the simultaneous removal of heavy-metal ions and organic pollutants. In these systems, photogenerated electrons in the C_B_ reduce toxic metal species, such as Cr(VI). In contrast, photogenerated h^+^ and ROS oxidize co-existing organic contaminants [174]. The open [Bi_2_O_2_]^2+^ layers in BiOX facilitate efficient separation of photo-induced e and h^+^, whereas heterojunction formation (e.g., BiOBr/BiOI, NaBiO_3_/BiOCl, BiOCl/BiNbO_4_/TiO_2_, BiOX/metal) accelerates interfacial charge transfer. Such dual-function systems are particularly relevant for complex industrial effluents containing both organic and inorganic contaminants, where BBPs can simultaneously achieve decolorization, TOC reduction, and metal detoxification under visible-light irradiation [175]. As demonstrated by Zhu et al., Bi-containing photoanodes (e.g., CuBi_2_O_4_/TiO_2_) primarily function as visible-light-driven electron generators and transport media. At the same time, cathodic Fe-based components serve as the dominant reduction sites for Cr(VI) → Cr(III) conversion. The deliberate spatial separation of oxidation and reduction reactions, together with rapid electron transfer, high reduction kinetics, low metal leaching, and good operational stability in real wastewater matrices, highlights the practical applicability of Bi-based PEC architectures for heavy-metal reduction in advanced water treatment systems [176].

Building on their effectiveness in Cr(VI) reduction, Bi-based photo(electro)catalysts have been increasingly explored for the reductive transformation of Cu^2+^ ions, benefiting from similar charge-separation and electron-delivery mechanisms under visible-light irradiation. Zhang et al. systematically demonstrated that a Bi_2_O_3_/C/TiO_2_ p–n heterojunction photocatalyst exhibited highly efficient reduction of Cu^2+^ ions in aqueous systems. By integrating Bi_2_O_3_ with TiO_2_ and introducing in situ-derived carbon bonds, the composite achieved markedly enhanced light absorption, charge separation, and electron transfer. Under visible-light irradiation, the optimized 0.1Bi_2_O_3_/0.3C/1.5TiO_2_ catalyst removed 97.39% of Cu^2+^ within 120 min, with XPS analysis confirming that Cu^2+^ was predominantly reduced to metallic Cu^0^ (85.64%), alongside Cu^+^ species. Mechanistic investigations revealed that the internal electric field formed at the Bi_2_O_3_/TiO_2_ p–n junction, coupled with conductive carbon bonds, effectively directed photogenerated electrons toward surface-adsorbed Cu^2+^ ions, thereby suppressing e-h recombination and favoring multi-electron reduction pathways. Importantly, the catalyst maintained high Cu^2+^ reduction efficiency (>85%) after multiple cycles and showed strong resistance to interference from coexisting metal ions (Ni^2+^, Pb^2+^, Hg^2+^, Cd^2+^), highlighting the robustness of Bi-based heterostructures for practical heavy-metal remediation applications [177].

Beyond Cu^2+^ reduction, Bi-based photo(electro)catalytic systems have also been extended to the remediation of other toxic heavy metals, particularly Hg(II), where the reduction mechanism and the role of Bi-based materials differ markedly, as demonstrated by Chang et al., who developed a visible-light-responsive S-scheme Bi_2_O_3_/Bi/g-C_3_N_4_ heterojunction with nitrogen and O_v_ for efficient Hg(II) reduction. The optimized catalyst achieved 99.95% Hg(II) removal within 120 min, with ~91% of the removal attributed to photocatalytic reduction rather than adsorption. XPS analysis confirmed the reduction of Hg(II) to Hg(0) (69.2%) and Hg(I) (21.8%), demonstrating that reduction was the dominant pathway. The enhanced activity originated from the S-scheme charge transfer, metallic Bi-mediated electron transport, and defect-induced carrier migration. The system also showed excellent durability, retaining >96% efficiency after five cycles, highlighting the strong potential of Bi-based photocatalysts for mercury remediation in water [178].

#### 8.1.4. Gas-Phase Pollutant Removal and Broader Environmental Remediation

Nitrogen oxides (NO_x_), primarily emitted from industrial processes, transportation, and energy production, are major atmospheric pollutants that pose serious risks to human health and ecosystems. NO and NO_2_ participate in atmospheric reactions that generate secondary pollutants, such as acid rain, photochemical smog, and ground-level ozone, thereby exacerbating air quality deterioration. Although physical adsorption, biofiltration, and thermal catalytic reduction have been explored for NO_x_ abatement [179], these methods often suffer from high energy consumption, secondary pollution, or limited efficiency under ambient conditions, underscoring the need for more sustainable removal strategies.

A 2D Bi_2_O_2_CO_3_/Bi_4_O_5_Br_2_ heterostructured photocatalyst synthesized via a one-step hydrothermal route proved an efficient system for NO_x_ removal under solar irradiation. The face-to-face coupling of ultrathin nanosheets formed a direct Z-scheme heterojunction, in which photogenerated electrons in Bi_2_O_2_CO_3_ recombine with h^+^ in Bi_4_O_5_Br_2_, preserving strongly reducing electrons in Bi_4_O_5_Br_2_ and highly oxidizing h^+^ in Bi_2_O_2_CO_3_. Consequently, the optimized 30 wt% composite achieved a NO_x_ removal efficiency of ~53.2%, significantly higher than the individual components. PEC analyses confirmed reduced recombinations and interfacial transfer. At the same time, ESR and trapping experiments identified **^•^**O_2_^−^ and **^•^**OH as the dominant reactive species, with electrons playing a key role in NO oxidation. In situ Fourier transform infrared spectroscopy (FTIR) results further revealed the efficient conversion of NO to surface nitrites and nitrates with suppressed NO_2_ accumulation, highlighting the advantage of Z-scheme charge management for gas-phase NO_x_ remediation [180].

A coral-like BiVO_4_/g-C_3_N_4_ photocatalyst prepared via an in-situ calcination method demonstrated to be an effective visible-light system for gas-phase volatile organic carbon (VOC) removal. The intimate interfacial coupling between BiVO_4_ and g-C_3_N_4_ forms a direct Z-scheme heterojunction while maintaining strong redox potentials. The optimized composite exhibited a toluene degradation rate constant and mineralization efficiency 3.2 and 4.5 times higher, respectively, than those of pristine g-C_3_N_4_ under visible-light irradiation. ESR and photoluminescence analyses confirmed that **^•^**O_2_^−^, **^•^**OH, and photogenerated h^+^ are the dominant reactive species, enabling efficient oxidation and mineralization of toluene via the Z-scheme pathway [181].

### 8.2. Performance in Real and Complex Matrices

Alulema-Pullupaxi et al. reported that the PEC performance observed in real water and wastewater matrices often differs substantially from that obtained in laboratory studies conducted using simplified electrolytes. While most PEC investigations are performed in model solutions under controlled pH and electrolyte conditions, real wastewater contains complex matrices composed of inorganic ions, organic matter, and variable chemical compositions that significantly influence degradation efficiency. The authors noted that these matrix components can compete with target pollutants for reactive species, alter adsorption behavior, and reduce mineralization efficiency, while maintaining high photocurrent responses. Consequently, the discrepancy between laboratory-scale PEC performance and that achieved in real wastewater treatment has been identified as a major challenge for practical application and scale-up, underscoring the need for more studies under realistic operating conditions [182].

A representative example of matrix-controlled electrolyte chemistry is provided by bicarbonate/carbonate systems: Zhou et al. demonstrated that for BiVO_4_ photoanodes, bicarbonate anions act as efficient reaction mediators that promote rapid interfacial h^+^ extraction and strongly suppress surface recombination, thereby enhancing PEC performance. However, the long-term stability of BiVO_4_ shown is highly dependent on buffering, as unbuffered bicarbonate electrolytes induced measurable leaching of Bi and V due to alkaline chemical attack. In contrast, near-neutral buffered bicarbonate enabled stable operation with minimal corrosion [183].

Lado Ribeiro et al. reported that common inorganic ions present in real water matrices, including chloride, sulfate, and nitrate, can strongly influence oxidation pathways by scavenging highly reactive radicals or promoting the formation of secondary, less reactive radical species. In particular, chloride and sulfate were shown to divert oxidation toward halogen- and sulfate-based radicals, often reducing mineralization efficiency and altering transformation pathways. At the same time, nitrate may inhibit or promote degradation depending on irradiation conditions. Such matrix effects are widely observed across advanced oxidation technologies and contribute to discrepancies between pollutant degradation and mineralization performance in complex waters. The authors emphasized that prolonged operation and surface interactions in heterogeneous systems further exacerbate these effects, underscoring the importance of accounting for water-matrix composition when evaluating oxidation processes under realistic conditions [184].

Consequently, application-relevant benchmarking of Bi-based PEC materials should extend beyond single-pollutant tests performed in simplified electrolytes. As discussed, meaningful evaluation requires the inclusion of more realistic water matrices, such as mixed inorganic ion systems and water containing natural organic matter or related background constituents, and validation with real water or wastewater samples. In addition to reporting pollutant conversion, it emphasizes the importance of assessing mineralization efficiency (e.g., through TOC or COD measurements where feasible), along with stability and durability indicators, including long-term operation and potential material degradation or leaching. Such comprehensive benchmarking under representative solution-chemistry conditions is necessary to reflect practical treatment scenarios better and to bridge the gap between laboratory-scale PEC studies and real-world applications [7].

## 9. Summary and Future Perspective

Bismuth-based semiconductors have established themselves as auspicious materials for solar-driven PC and PEC owing to their favorable band structures, strong visible-light absorption, and structural versatility. Advances in synthesis strategies, including hydrothermal, sol–gel, spray pyrolysis, electrodeposition, and microwave-assisted methods, now enable precise control over morphology, crystallinity, and defect chemistry, directly enhancing catalytic reactivity. Performance-enhancing modifications such as elemental doping, oxygen-vacancy engineering, and rational heterojunction design (Type-II, p–n junctions, Z-scheme and S-scheme) further address the limitations of pristine Bi-based materials by improving band alignment and strengthening redox activity. These engineered systems consistently provide good photocurrent densities and high degradation efficiencies across a broad spectrum of pollutants. Under optimized conditions, many systems achieve pollutant removal exceeding 90% with higher mineralization. Their applicability extends to real wastewater, disinfection, cyanotoxin removal, and gas-phase remediation, demonstrating good stability and resilience under practical conditions. Overall, the convergence of advanced synthesis, defect modulation, and interfacial engineering has positioned BBPs as promising candidates for next-generation environmental purification. Continued efforts to optimize long-term stability, mechanistic understanding, and scalable fabrication will be key to transitioning these materials from laboratory demonstrations to real-world water and air treatment technologies. 

Despite significant progress, several critical challenges must be addressed to advance bismuth-based photo(electro)catalysts toward practical environmental remediation.

1. A central knowledge gap remained, the incomplete mechanistic understanding of structure–activity relationships, particularly the dynamic roles of O_v_, dopants, and heterojunction interfaces under operating conditions. Although these features are widely recognized as key performance regulators, their real-time evolution during PEC operation, such as vacancy migration, interfacial band bending, and charge recombination, remains poorly resolved. Addressing this challenge will require the systematic integration of operando and ultrafast characterization techniques with theoretical modeling to correlate dynamic structural changes with catalytic function directly.
2. Material stability constitutes another major bottleneck. Many Bi-based systems, especially Bi_2_O_3_ and bismuth chalcogenides, suffer from photocorrosion, phase transformation, and defect annihilation during prolonged operation, with degradation further accelerated in complex water matrices containing inorganic ions, natural organic matter, and radical scavengers. Future material design strategies should therefore prioritize stability-oriented approaches, including defect-stabilizing cocatalysts, corrosion-resistant heterostructures, protective surface coatings, and dynamically reconstructed or self-healing interfaces that can sustain long-term operation.
3. PEC enhancement strategies should be developed based on a clear identification of material-specific failure mechanisms rather than empirical photocurrent improvement alone. Aligning modification approaches, such as heterojunction construction, cocatalyst loading, protective layers, or hole-transport layers with the dominant degradation pathways (e.g., carrier accumulation, photocorrosion, or lattice dissolution) is essential to improve charge management and long-term stability simultaneously.
4. From a manufacturing perspective, reproducible and scalable synthesis of bismuth-based photo(electro)catalysts remained unresolved. While hydrothermal, sol–gel, and microwave-assisted methods offer excellent laboratory-level control over morphology and defects, their industrial translation is constrained by batch variability, energy intensity, and limited yield consistency. Progress toward practical deployment will depend on scalable synthesis platforms, such as continuous-flow hydrothermal processing, spray pyrolysis, plasma-assisted methods, and green precursor chemistries, that enable reliable defect and interface engineering.
5. At the device and system level, challenges including poor film adhesion, non-uniform thickness, unstable semiconductor substrate interfaces, and limited durability continue to restrict PEC performance. Promising future directions include the development of three-dimensional nanostructured photoelectrodes, conductive scaffold-supported catalysts, membrane electrode assemblies, and tandem PEC–advanced oxidation process (AOP) configurations. Integration of PEC systems with complementary treatment technologies, such as peroxymonosulfate activation, ozonation, or biological post-treatment, also represents a viable pathway for enhancing treatment efficiency in complex wastewater.
6. From an application standpoint, most studies remain focused on model pollutants at unrealistically high concentrations. In contrast, real environmental systems involve complex mixtures of trace-level contaminants, microorganisms, and resistance genes that can severely suppress PEC performance. Future research should therefore prioritize testing under environmentally relevant conditions, including real wastewater matrices and long-term operation. Beyond pollutant degradation, functional expansion, such as photocatalytic antimicrobial inactivation, remains underdeveloped and requires improved control over band energetics, electron-transport pathways, and selective cocatalyst design to enable efficient multi-electron reactions.
7. Finally, environmental safety, techno-economic feasibility, and life-cycle impacts must be integrated early in materials development. Comprehensive life-cycle assessments, ecotoxicological evaluations, catalyst regeneration studies, and cost-per-treatment analyses are essential for identifying scalable, safe, and economically viable PEC remediation strategies.

Overall, future progress in bismuth-based photo(electro)catalysts will depend on moving beyond isolated material optimization toward integrated system design, combining mechanistic insight, stability engineering, scalable manufacturing, realistic performance evaluation, and reactor-level integration. Such a holistic approach is essential for translating Bi-based photo(electro)catalysts from laboratory innovation to practical technologies for environmental remediation and sustainable energy conversion.

## Figures and Tables

**Table 1:** Summary of bismuth-based materials.

Class	Band Gap (eV)	Carrier Type	Stability	Typical Applications
Bismuth Oxides	2–3.9	p-type	Polymorph-dependent; α-Bi_2_O_3_ stable at ambient conditions, while β- and γ-phases are metastable; δ-Bi_2_O_3_ stabilized at high temperature or via defect/dopant engineering.	Photocatalytic degradation of organic pollutants (ROS-mediated).
Bismuth Chalcogenides	0.3–1.2	n-type	Phase-dependent; narrow band gaps enable strong visible-light absorption but are prone to photocorrosion under prolonged operation.	Visible-light-driven photo(electro)catalysis; pollutant degradation; charge-dynamics tuning via doping, surface modification, and heterojunctions.
Bismuth Oxychalcogenides	~0.8–1.27	n-type	High environmental stability due to layered structures and robust Bi–O frameworks.	Visible-light PEC applications; heterojunction and defect engineering to enhance performance.
Bismuth Oxyhalides	1.9–3.9	n/p-type	Layered structure prolongs carrier lifetime; BiOI shows improved PEC stability, though recombination remains a limiting factor.	Photocatalytic pollutant degradation; PEC oxidation; performance enhancement via doping and heterojunctions.
Bismuth-Based Perovskite-like Oxides	2.0–2.2	n/p-type	Structurally stable; intrinsic ferroelectric polarization promotes charge separation and suppresses recombination.	Visible-light PEC applications; enhanced via doping (e.g., La, Mn), heterojunctions, and nanostructuring.
Bismuth-Based Metal Oxides	~2.4–2.8	n-type	Generally stable; BiVO_4_ exhibits high PEC stability, Bi_2_MoO_6_ and Bi_2_WO_6_ suffer from charge recombination.	PC pollutant degradation; PEC photoanodes, including water splitting and CO_2_ reduction.

**Table 2:** Comparison of synthesis strategies for Bi-based materials.

Method	Advantages	Limitations	Scalability	Typical Features	Outcome
Solid-state reaction	Simple, solvent-free, low cost; high crystallinity; bulk synthesis	High temperature (≈400–800 °C); grain growth; poor morphology/defect control	High	Micron-sized, dense polycrystalline particles	Phase-pure oxides; low surface area; generally unsuitable for high-performance PEC
Co-precipitation	Low-temperature, simple, good composition control	Particle aggregation, limited facet/defect control, pH-sensitive	Moderate–High	Aggregated nanoparticles, plate-like particles, BiOX nanosheets	Suitable for PC, PEC requires further structuring or film fabrication
Sol–gel	High homogeneity, tunable size and defects, band-structure control	Multi-step, long gelation/drying, cracking; scale-up challenges	Moderate	Nanoparticles, porous xerogels, networked or flower-like structures	Good visible-light PC, moderate PEC relevance after film processing
Hydro/solvothermal	Excellent phase, facet, and morphology control, high crystallinity at moderate temperature	Long reaction times, batch process, limited reactor volume	Moderate	Nanosheets, nanoplates, nanorods, hierarchical architectures	Highly effective PC and PEC with enhanced charge separation
Microwave-assisted hydro/solvothermal	Ultrafast, energy-efficient, rapid crystallization, defect-rich products	Limited reactor size, equipment cost, and uniformity control	Low–Moderate	Fine nanoparticles, mixed phases, quantum dots	Strong photocurrent response, promising for advanced PEC systems
Spray pyrolysis	Continuous deposition, thickness and stoichiometry control, industrial relevance	Low material utilization, narrow temperature window, and post-annealing are often required.	High	Dense or porous thin films; granular grains; hollow microspheres	Robust PEC photoelectrodes with good adhesion and stability
Electrodeposition	Low temperature, precise thickness/morphology control, direct substrate growth	Requires conductive substrates, parameter-sensitive, and post-conversion is often needed	High	Nanowires, nanoflakes, flower-like films on FTO/ITO	Excellent charge transport, ideal for PEC degradation

**Table 3:** The degradation efficiencies of bismuth-based photocatalysts.

Materials	Light Source	Pollutant	Key Performance	Main Active Species
TS/BiVO_4_	Natural sunlight	RhB	Nearly complete RhB degradation within 60 min; superior activity vs. TS and BiVO_4_	^•^O_2_^−^, ^•^OH, and h^+^
Bi_2_O_3_/Bi_2_S_3_/MoS_2_	Simulated solar light	MB	O_2_ evolution rate of 529.1 μmol h^−1^ g^−1^; ~90% MB degradation	^•^O_2_^−^ and ^•^OH
Bi(0)-doped BiOCl_0.875_Br_0.125_	Solar irradiation	SMX, CBZ, VLX, BZF	Highest degradation performance among tested films; outperformed TiO_2_	ion-exchange-assisted adsorption followed by oxidation
Bi_2_Se_3_	Visible light	MO and Cr(VI)	81.2% MO degradation in 240 min (k = 0.0038 min^−1^); 88.4% Cr(VI) reduction in 60 min (k = 0.007 min^−1^)	^•^OH and ^•^O_2_^−^ for MO degradation; electrons reduce Cr(VI)
BSO	Ultraviolet light	TC	95.79% degradation in 80 min; k = 0.0361 min^−1^; >80% efficiency after four cycles	Photocatalytic oxidation with structural transformation to Bi_2_O_2_CO_3_

**Table 4:** Pollutant degradation efficiencies of different Bi-based PEC systems.

Material	Pollutant	Bias (V)	Light Source	Conc. (ppm)	Electrolyte	Deg. (%)	Time (min)	Ref.
Bi_2_O_3_/TiO_2_	RhB	0.5 vs. Ag/AgCl	150 W lamp	20	0.1 M Na_2_SO_4_	100	40	[143]
Ag-BiVO_4_/BiOI	DFS	1.0 vs. Ag/AgCl	100 W Xe lamp	10	0.1 M Na_2_SO_4_	92	120	[144]
TiO_2_-BiVO_4_-PI	BPA	1.0 vs. SCE	300 W Xe lamp	5	H_2_O	93.5	120	[145]
Bi/Bi_2_S_3_/α-MoO_3_	TC	1.0 vs. Ag/AgCl	300 W Xe lamp	30	0.1 M NaCl	85.8	60	[146]
n-MnO_2_/BiOI	TC	1.0 vs. Ag/AgCl	300 W Xe lamp	30	0.1 M NaCl	95.8	120	[147]
PDISA/Bi_2_WO_6_	TC	1.2 vs. RHE	300 W Xe lamp	20	0.5 M Na_2_SO_4_	98.4	90	[148]
ZnO/Bi_3_TaO_7_	Cip	N/A-	100 W Xe lamp	5	0.1 M Na_2_SO_4_	98	180	[123]
BFO/BVO	Cip	2.0 vs. Ag/AgCl	100 W Xenon lamp	5	0.1 M Na_2_SO_4_	80.3	240	[149]
Bi_4_Ti_3_O_12_/TiO_2_	TC	1.0 external cell volt.	300 W Xe lamp	20	0.05 M Na_2_SO_4_	99.7	75	[84]
Bi_2_S_3_/Bi_2_MoO_6_/TiO_2_	MG	0.6 vs. Ag/AgCl	100 mW/cm^2^ Xe lamp	10	0.5 M Na_2_SO_4_	86.69	120	[150]
Zr:BiVO_4_@Bi_2_S_3_/CoS	TCH	0.5 vs. SCE	100 mW/cm^2^ Xe lamp	20	0.1 M Na_2_SO_4_	94	60	[151]
BiVO_4_	BZP	1.0 vs. Ag/AgCl	300 W Xe lamp	2	1.5 mM Na_2_SO_3_	92.3	90	[152]
Bi_2_WO_6_	RhB	10 external cell volt.	300 W Xe lamp	30	0.5 M Na_2_SO_4_	100	30	[153]
(SDS-Ni-BOC/MGF)	BPA	2.0 two-electrode	300 W Xe lamp	5	0.01 M Na_2_SO_4_	100	30	[154]
Ag_3_PO_4_/BiVO_4_	NOR	0.5 vs. SCE	300 W Xe lamp	5	10 mM NaClO_4_	100	90	[155]

RhB = Rhodamine B dye, DFS = Diclofenac sodium, TCH = tetracycline hydrochloride, Cip = ciprofloxacin, MG = malachite green, BZP = Benzophenone-3, BPA = Bisphenol A, NOR = Norfloxacin, (N/A = not available).

## Data Availability

No new data were created or analyzed in this study. Data sharing is not applicable to this article.
